# FDA-Approved Drugs Containing Amide Functionality in the Last Five Years (2021–2025): Pharmaceutical Use, Trends and Synthetic Approaches

**DOI:** 10.3390/medicines13030022

**Published:** 2026-07-07

**Authors:** Davide Benedetto Tiz

**Affiliations:** Laboratory of Molecular Logic Gates, Department of Chemistry, Faculty of Science, University of Malta, MSD 2080 Msida, Malta; davide.benedetto-tiz@um.edu.mt

**Keywords:** amide, nitrogen, oxygen, hydrogen bond, rigidity, drugs, FDA

## Abstract

The amide functional group remains a cornerstone of medicinal chemistry, serving as an indispensable scaffold in the design of modern therapeutics. This review presents an analysis of FDA-approved drugs (small molecules and peptides with MW < 1300 Da) containing amide functionality between 2021 and 2025, highlighting its continued and evolving role in addressing contemporary medical challenges. An analysis of these novel therapeutics reveals the remarkable functional versatility of the amide bond. In antiviral agents like nirmatrelvir (Paxlovid^®^), amides form the structural backbone of peptidomimetics, enabling high-affinity binding to a viral protease. In precision oncology, as seen with adagrasib (Krazati^®^), the amide acts as a critical, metabolically stable linker that positions a covalent warhead for selective inhibition of a mutant kinase. This analysis underscores that amide’s unique combination of planarity, resonance stabilization, and capacity for robust hydrogen bonding continues to make it an essential element in the medicinal chemist’s toolkit, underpinning the development of next-generation therapeutics across oncology, infectious diseases, and neurology. To provide a practical framework for drug discovery, the synthetic routes for each drug are detailed, with particular emphasis placed on the key amide-forming strategies employed.

## 1. Introduction

The amide functional group plays a pivotal role in the composition of biomolecules, including many clinically approved drugs. Amide bonds are found in many natural compounds and popular drugs, which makes this functional group one of the most abundant in organic chemistry. Chemically speaking, an amide bond is a covalent linkage formed between a carboxyl group and an amino group, and it is the key structural feature of peptides and proteins. Structurally, in the case of a secondary amide, the bond consists of a carbonyl carbon (C=O) bonded to a nitrogen atom (–NH–), giving the characteristic of a –CO–NH– linkage. In primary amides, the nitrogen is bonded to the carbonyl carbon and two hydrogen atoms. In tertiary amides, the nitrogen is bonded to the carbonyl carbon and two alkyl/aryl groups (with no hydrogens). Over the past five years, significant advancements have been made in the activation of amides through complementary mechanisms that ultimately depend on ground-state destabilization of the amide linkage, despite the fact that typical amides are planar and the amide N–C(O) bond is notoriously difficult to break due to n_N_→π^⁎^C=O resonance [1].

**Figure 1:**
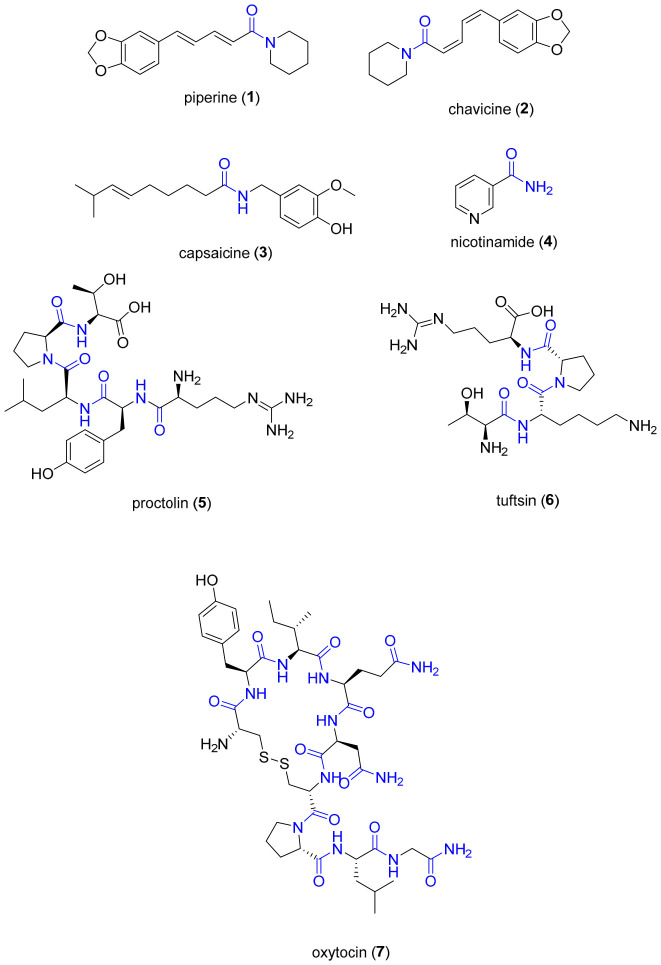
Chemical structure of natural compounds containing the amide functional group. See text for more details. The amide (cyclic or acyclic) functional group is depicted in blue.

Among natural compounds, compounds with amide function exhibit a wide spectrum of biological action. The pungency of black pepper, for example, is generated by the alkaloid piperine and its isomer chavicine [2,3] (with both containing cyclic amide groups **1** and **2**; Figure 1). Capsaicin (**3**; Figure 1) is the principal active component of chili peppers and gives them their “spicy” or “hot” flavor [4,5]. Nicotinamide (**4**; Figure 1) is a naturally occurring form of Vitamin B3, essential for metabolic processes [6,7]. Another class containing amide bonds is represented by peptides. Peptides are formed through amide bonds, also known as peptide bonds, which are the fundamental chemical linkages holding amino acids together. An amide bond is created when the carboxyl group (–COOH) of one amino acid reacts with the amino group (–NH_2_) of another, releasing a molecule of water in a condensation reaction [8]. This bond gives peptides their characteristic backbone structure, providing both stability and flexibility [9]. Because the amide bond is chemically robust yet capable of participating in hydrogen bonding, peptides can adopt the diverse three-dimensional shapes essential for their biological functions [10]. As a result, peptides are widespread in nature, forming the structural and functional basis of proteins involved in nearly all biological processes, including enzymes, hormones, signaling molecules, and structural components of cells [11,12]. The unique properties of the amide bond, its planarity, partial double-bond character, and resistance to spontaneous breakdown explain why peptides are so prevalent and reliable as molecular building blocks in living systems [10]. Among natural peptides is proctolin (**5**; Figure 1), a pentapeptide that is present in insects and crustaceans and acts as a neuromodulator and possibly as a neurohormone [13]. Found in mammals, tuftsin (**6**; Figure 1) is derived from immunoglobulin G (IgG) and plays an important role in immune response, especially in activating macrophages and neutrophils [14]. Oxytocin (**7**; Figure 1) is a peptide made up of nine amino acids. It is a reproductive hormone and is used to induce labor and control postpartum bleeding [15].

**Figure 2:**
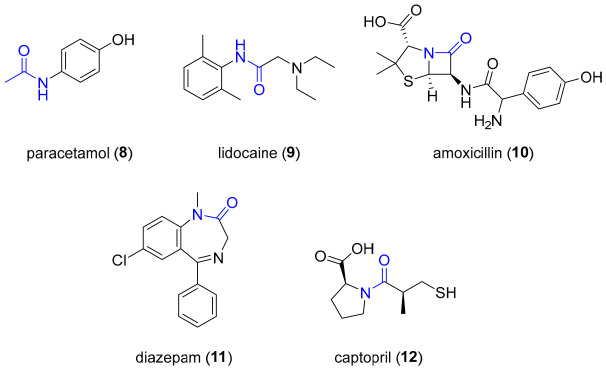
Pharmaceuticals containing the amide group. Amide groups shown in blue.

Moving to pharmaceuticals, the amide group improves stability, binding to target proteins, and mimicry of peptide bonds, helping these compounds act effectively as analgesics, antibiotics, anesthetics, CNS drugs, cardiovascular agents, and agrochemicals, as demonstrated by paracetamol (**8**), lidocaine (**9**), amoxicillin (**10**), diazepam (**11**), and captopril (**12**), see Figure 2.

Paracetamol (acetaminophen) acts primarily as a central nervous system (CNS) analgesic by reducing pain signaling in the brain and spinal cord [16,17]. Lidocaine is a widely used, effective local anesthetic and anti-inflammatory agent for rapid, short-term pain relief, commonly used in topical patches, creams, and IV infusions [18]. Amoxicillin is a widely used, bactericidal beta-lactam (four-membered lactam) antibiotic in the aminopenicillin family, designed to treat a broad spectrum of Gram-positive and Gram-negative bacteria [19]. Diazepam is a member of the benzodiazepine class, introduced in the 1960s. They are relatively safe drugs, in comparison with other types of drugs used to treat anxiety [20]. Captopril is an ACE inhibitor medication used to treat high blood pressure (hypertension), heart failure, and damage after a heart attack by relaxing blood vessels [21].

**Figure 3:**
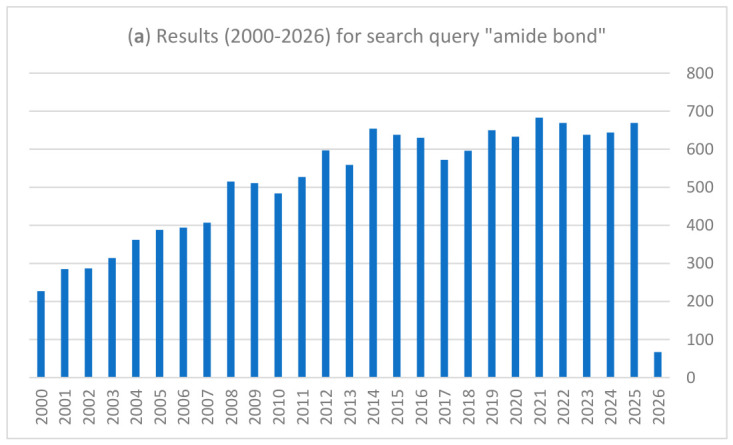

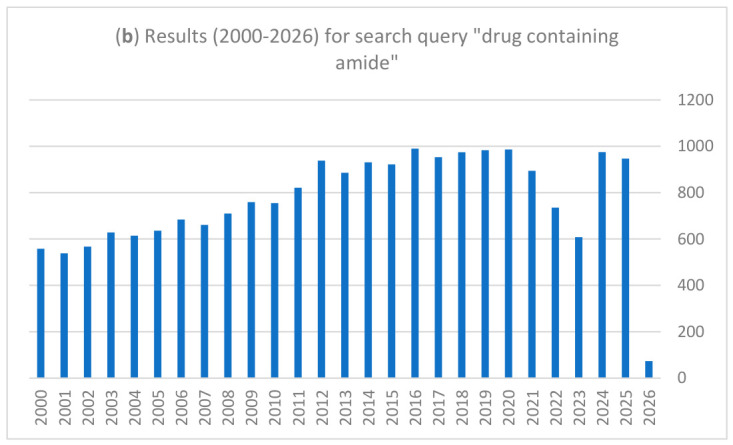
(**a**) Results for search query “amide bond” in years 2000–2026 [22]. (**b**) Results for search query “drug containing amide” in years 2000–2026. Data assessed on 28 January 2026.

Figure 3a illustrates a generally upward trend in the number of search results on PubMed for “amide bond” from the year 2000 through 2026, reaching a peak of around 700 results annually in the years 2014–2022 [22]. The visual trend is consistent with search results indicating a growing interest and the significant role of the amide bond in medicinal chemistry, drug development, and organic synthesis, particularly in recent decades. The formation of amide bonds is one of the most frequently performed reactions in both academic and industrial settings. Moreover, amide biocatalysis and its formation in flow is a rapidly advancing, high-impact field that merges the high selectivity of enzymes with the superior process engineering of continuous-flow chemistry, and many reviews have covered this topic widely [23,24,25]. Figure 3b [26] focuses specifically on drugs containing amide functionalities, allowing for a more targeted assessment of the relevance of amide bonds in medicinal chemistry and drug development. This refinement helps distinguish general chemical usage from trends directly related to therapeutic applications. It is interesting to note that a stable interest in this field and a decline in the period 2021–2023 are probably due to the COVID-19 pandemic.

## 2. FDA-Approved Drugs in 2021–2025 Containing Amide Group

In the next section, we will focus on the FDA-approved drugs (<1300 Da) that contain at least an amide functional group, excluding carbamates, sulfamides, and urea, despite these being closely related to amide. This topic has also been addressed in the review ‘A Perspective of the Amide Group Containing FDA Approved Anticancer Drugs from 2021–2022’ by Nasibullah [27]; however, our work differs by focusing on covering all the small molecules that contain an amide group in the period 2020–2025. When possible, the role of the amide linkage will be explained.

### 2.1. FDA-Approved Drugs in 2021 Containing Amide Group

In 2021, the FDA approved a total of 50 drugs, of which 36 were new chemical entities [28]. The approved drugs addressed a broad range of therapeutic areas, including oncology, infectious diseases, and rare disorders, reflecting ongoing efforts to meet unmet medical needs. The diversity of chemical structures among the NCEs, many of which contain key functional groups such as amides, underscores the importance of functional group chemistry in modern drug design and development. In the following sections, amide-containing small molecules will be presented.

#### 2.1.1. Pafolacianine

**Figure 4:**
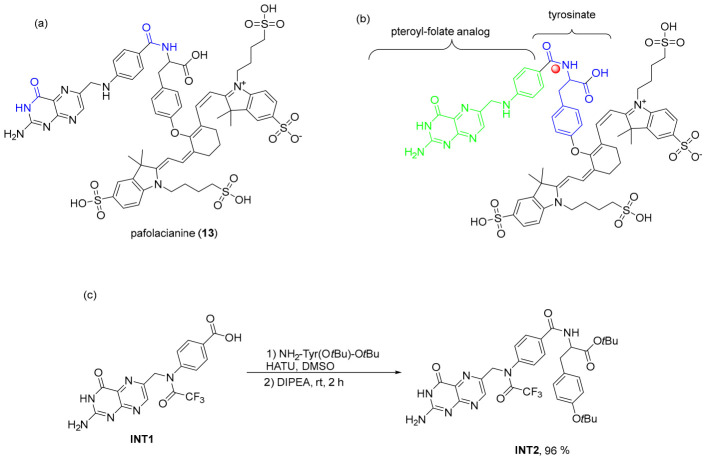
(**a**) Chemical structure of pafolacianine. (**b**) The folate analog (marked in green) is linked with a tyrosine amino acid (blue) through an amide linkage (red sphere). (**c**) Synthetic preparation of **INT2**, an intermediate in the synthesis of pafolacianine.

Pafolacianine (**13**, Figure 4a), marketed under the brand name Cytalux^®^, is a fluorescent optical imaging agent that was approved by the U.S. Food and Drug Administration (FDA) on 29 November 2021 for use in adult patients with ovarian cancer as an adjunct to help surgeons identify malignant lesions during surgery [29]. This approval marked the first FDA authorization of pafolacianine for intraoperative tumor detection, leveraging its ability to bind to folate receptors that are often overexpressed on ovarian cancer cells and to emit fluorescence under near-infrared light [30]. The decision was based on clinical evidence showing that the agent enabled the detection of additional cancerous tissue beyond what is visible with standard surgical inspection techniques, thereby potentially improving surgical outcomes [31]. The structure is a large, complex organic molecule composed of multiple aromatic and heteroaromatic ring systems linked by flexible aliphatic chains. Two amide functional groups are present, contributing to molecular rigidity, resonance stabilization, and hydrogen-bonding capability. A folate analog (on the left portion of the molecule) is linked to a tyrosine amino acid through an amide linkage (Figure 4b) [29]. The molecule also contains sulfonic acid groups, which introduce strong polarity and a negative charge, significantly enhancing aqueous solubility. Multiple heterocycles containing nitrogen atoms are incorporated, increasing the potential for specific biological interactions through hydrogen bonding and electrostatic forces. Phenyl and fused aromatic systems provide hydrophobic regions, creating an amphiphilic character with distinct polar and nonpolar domains. Overall, the chemical architecture reflects a balance between aromatic hydrophobic scaffolds and strongly polar, ionizable functional groups, a design well suited for biological targeting. The amide linker is introduced via a HATU/DIPEA coupling strategy (Figure 4c) starting from triflouroacetylpteroic acid as the starting material (**INT1**) and (O*t*Bu)-protected tyrosine, affording **INT2** in 96% yield [32].

#### 2.1.2. Asciminib

**Figure 5:**
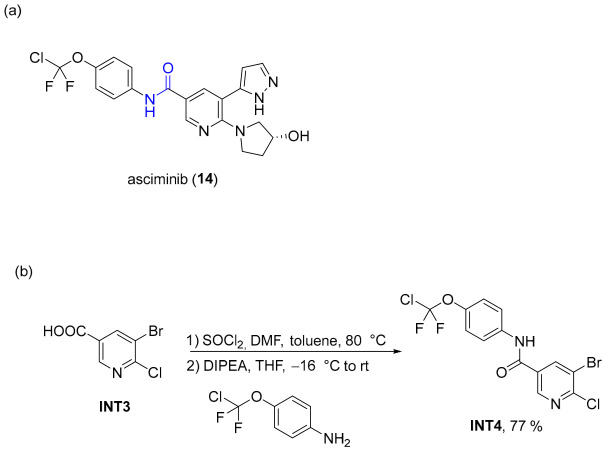
(**a**) Chemical structure of asciminib. (**b**) Synthetic preparation of INT4, an intermediate in the synthesis of asciminib.

Asciminib (**14**, brand name Scemblix^®^, Figure 5a) is an FDA-approved targeted therapy for Philadelphia chromosome-positive chronic myeloid leukemia (Ph+ CML) in the chronic phase. It was first granted accelerated approval by the FDA on 29 October 2021 for adult patients with Ph+ CML in chronic phase who had received two or more prior tyrosine kinase inhibitors, and for patients with the T315I mutation, which is associated with treatment resistance [33,34]. Asciminib represents a novel class of tyrosine kinase inhibitor that uniquely binds to the myristoyl pocket of the BCR-ABL1 fusion protein, a distinct mechanism compared with other TKIs that target the ATP binding site, thereby potentially overcoming resistance seen with earlier therapies [35]. Clinically, asciminib has demonstrated improved major molecular response rates and a favorable safety profile in randomized trials versus standard therapy, offering an important option for patients with difficult-to-treat CML [36]. Chemically, asciminib is a small-molecule, heteroaromatic compound containing multiple nitrogen-rich ring systems and one amide functional group, which contributes to its polarity and hydrogen-bonding capability. The structure incorporates a benzamide moiety that has been linked with enhanced potency and kinase selectivity [37]. Overall, asciminib exhibits a balanced amphiphilic character, combining polar functional groups for target recognition with hydrophobic scaffolding for effective pharmacokinetic behavior. The preparation of **INT4**, an intermediate in the synthesis of asciminib, is obtained via activation of carboxylic acid (**INT3**) using thionyl chloride and 4-(chlorodifluoromethoxy)aniline to give the amide building block in 77% yield [38].

#### 2.1.3. Avacopan

**Figure 6:**
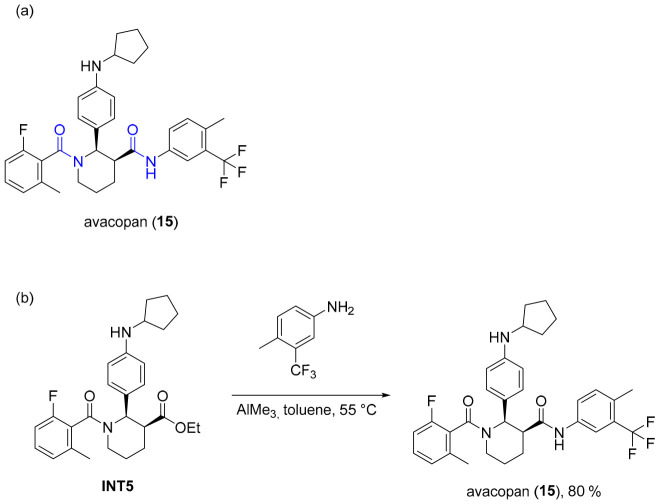
(**a**) Chemical structure of avacopan. (**b**) Final synthetic step for the preparation of avacopan.

Approved on 7 October 2021, avacopan (**15**, brand name Tavneos^®^, Figure 6a) is a small-molecule oral drug that selectively blocks the complement 5a receptor (C5aR), part of the immune system involved in inflammation. By antagonizing C5aR, it helps reduce neutrophil-mediated inflammation in small blood vessels [39,40]. It is used as an adjunctive treatment (added to standard immunosuppressive therapy, including glucocorticoids) for adults with ANCA-associated vasculitis [39,40]. Moving to its chemistry, the combination of a rigid core, aromatic hydrophobic regions, fluorinated substituents, and strategically placed amide groups (two) reflects a classic medicinal-chemistry design for a potent, selective, and orally active C5aR antagonist. The synthesis of avacopan (Figure 6b) is accomplished via coupling the ethyl ester (**INT5**) using AlMe_3_-mediated direct coupling with 4-methyl-5-trifluoromethylaniline to give the desired drug in good yield (80%) [32].

#### 2.1.4. Atogepant

**Figure 7:**
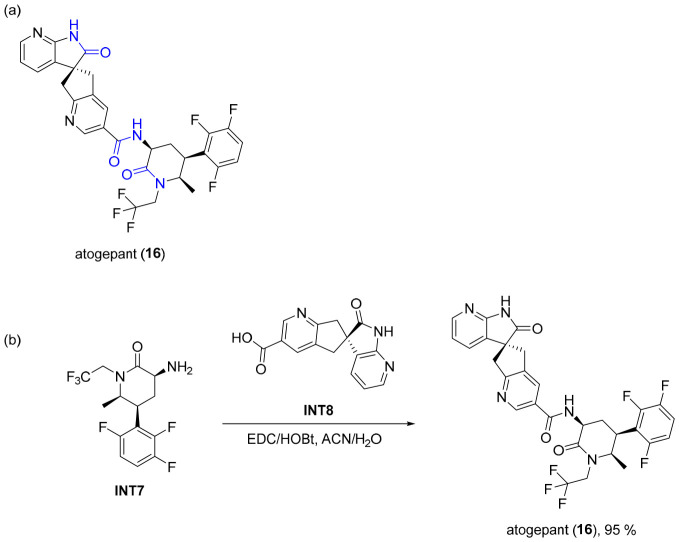
(**a**) Chemical structure of atogepant. (**b**) Final synthetic step for the preparation of atogepant.

Atogepant (**16**, brand names Qulipta^®^ and Aquipta^®^, Figure 7a) is an orally administered, small-molecule calcitonin gene-related peptide (CGRP) receptor antagonist in the “gepant” class, developed primarily for preventive treatment of migraine headaches in adults [41,42]. It works by blocking the CGRP receptor, reducing CGRP-mediated neurogenic inflammation, vasodilation, and nociceptive signaling that contribute to migraine pathophysiology. This targeted mechanism helps lower the frequency of migraine days when taken once daily in doses such as 10 mg, 30 mg, or 60 mg tablets [43,44]. The structure is a complex, stereochemically defined small molecule built around multiple heterocycles and three amide linkages, indicating a highly optimized, target-specific design. A rigid fused heteroaromatic core on the left likely serves as a key binding motif, while the central heteroaryl and amide connectors balance rigidity with controlled flexibility. The fluorinated aromatic ring and CF_3_/difluoro substituents on the right enhance lipophilicity, metabolic stability, and binding affinity. Overall, the molecule shows a deliberate balance of polarity, rigidity, and a three-dimensional shape typical of a late-stage medicinal chemistry lead or clinical candidate. The final step of preparation for atogepant (Figure 7b) involves a condensation between amine (**INT7**) and carboxylic acid **INT8** via the EDC/HOBt coupling reagent [32].

#### 2.1.5. Mobocertinib

**Figure 8:**
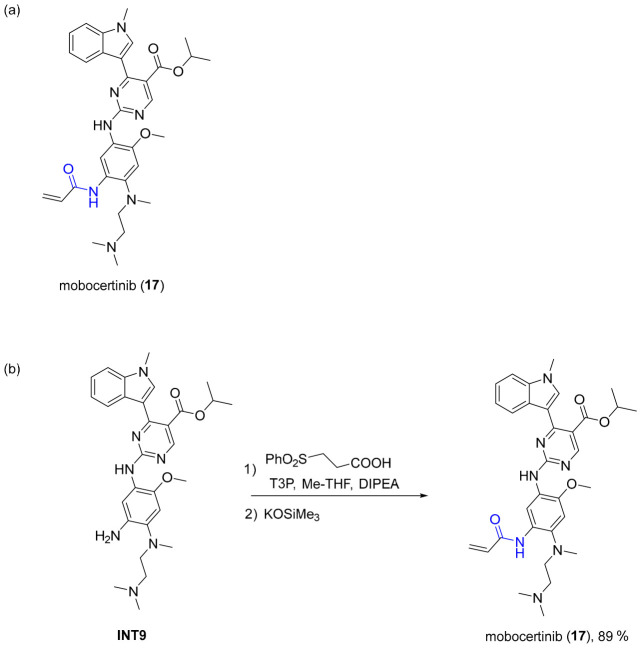
(**a**) Chemical structure of mobocertinib. (**b**) Final synthetic step for the preparation of mobocertinib.

Approved by the FDA on 15 September 2021, mobocertinib (**17**, brand name Exkivity^®^, Figure 8a) is an oral, small-molecule tyrosine kinase inhibitor designed to selectively target epidermal growth factor receptor (EGFR) exon 20 insertion mutations in non-small-cell lung cancer (NSCLC), a subset that is poorly responsive to other EGFR inhibitors [45]. It covalently and irreversibly inhibits mutant EGFR (and related family kinases) to block signaling that drives tumor growth. Mobocertinib was granted accelerated approval by the FDA and is indicated for adults with locally advanced or metastatic EGFR exon 20 insertion-positive NSCLC after platinum-based chemotherapy [46]. In pivotal trials, it showed meaningful antitumor activity with objective response rates in previously treated patients and median progression-free survival in the range seen for targeted therapies, although confirmatory trials are required to maintain approval. Common adverse effects include diarrhea, rash, nausea, and other gastrointestinal and skin toxicities [47]. The most functionally significant feature of this structure is the acrylamide warhead, which is a hallmark of covalent kinase inhibitors. The α,β-unsaturated carbonyl acts as a Michael acceptor, enabling selective, irreversible covalent bonding with a nucleophilic cysteine residue in the target protein (classically in EGFR-family kinases) [48]. This transforms the molecule from a reversible binder into a targeted covalent inhibitor, increasing potency, duration of action, and target residence time. Importantly, the acrylamide is positioned at the end of a flexible linker, allowing precise geometric alignment with the cysteine in the binding pocket, which improves selectivity and reduces off-target reactivity. Mobocertinib (Figure 8b) was synthesized in an 89% yield starting from **INT9** and 3-(phenylsulfonyl)propanoic acid. This process was driven by propylphosphonic anhydride (T3P)-mediated coupling, followed by the elimination of the phenylsulfonyl group using potassium trimethylsilanolate [45].

#### 2.1.6. Odevixibat

**Figure 9:**
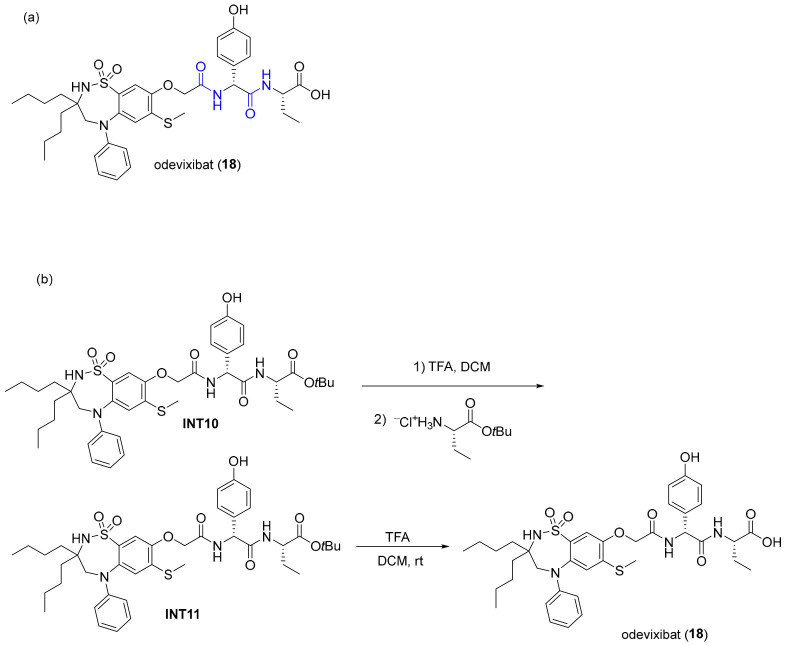
(**a**) Chemical structure of odevixibat. (**b**) Final steps for the synthesis of odevixibat (**18**).

Odevixibat (**18**, brand name Bylvay^®^, Figure 9a) is an oral ileal bile acid transporter (IBAT) inhibitor developed for the treatment of rare pediatric cholestatic liver diseases [49]. By blocking bile acid reabsorption in the terminal ileum, it reduces systemic bile acid levels, which are responsible for liver injury and severe pruritus in these conditions [50,51]. In the United States, odevixibat was approved by the FDA in July 2021 for the treatment of pruritus in patients aged three months and older with progressive familial intrahepatic cholestasis (PFIC) [49]. This approval was based on clinical trials demonstrating significant reductions in serum bile acids and improvements in itch severity. Its chemical structure is characterized by a central benzothiadiazepine core linked to a peptide-like side chain (two amide bonds are counted). Odevixibat is a relatively large, flexible molecule with a mixed polarity profile. It contains multiple polar functionalities (amide, carboxylic acid, heteroatoms capable of hydrogen bonding) alongside a substantial hydrophobic component. This means it is neither purely hydrophilic nor purely lipophilic. Regarding the synthetic preparation (Figure 9b), the deprotection of tert-butyl ester **INT10** using trifluoroacetic acid (TFA) in dichloromethane, followed by a second peptide coupling with tert-butyl (*S*)-2-aminobutanoate, yielded dipeptide **INT11**. Subsequent acidic hydrolysis of intermediate INT11 then afforded the target compound, odevixibat (**18**) [32].

#### 2.1.7. Belumosudil

**Figure 10:**
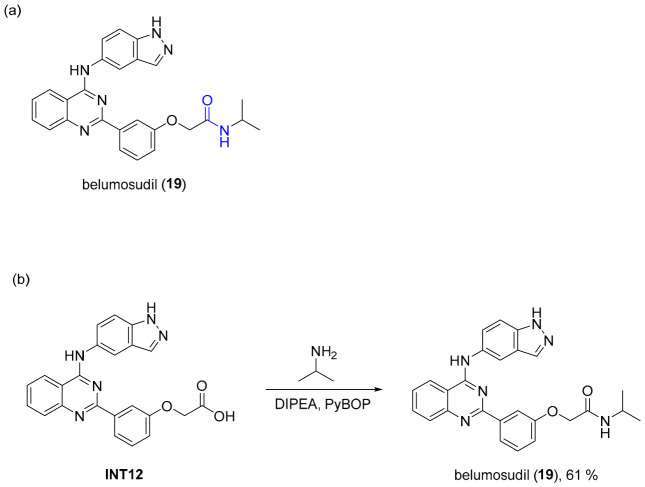
(**a**) Chemical structure of belumosudil. (**b**) Final step for the synthesis of belumosudil (**19**).

Approved on 16 July 2021, belumosudil (**19**, brand name Rezurock^®^, Figure 10a) is an oral small-molecule drug approved by the U.S. FDA for the treatment of adults and pediatric patients 12 years of age and older with chronic graft-versus-host disease (cGVHD) after failure of at least two prior lines of systemic therapy [52,53]. It works by selectively inhibiting Rho-associated coiled-coil-containing protein kinase 2 (ROCK2), a signaling enzyme involved in immune dysregulation and fibrosis [54]. By targeting ROCK2, belumosudil helps rebalance pro-inflammatory and regulatory immune pathways while also reducing fibrotic processes that contribute to organ damage in cGVHD [55]. Clinical studies showed meaningful overall response rates across multiple affected organs, with many patients experiencing durable responses and improvements in symptoms [56]. The drug is generally well tolerated, with common side effects including fatigue, diarrhea, nausea, cough, and infections [57], making it a targeted therapeutic option for patients with difficult-to-treat chronic GVHD. The molecule is a polycyclic heteroaromatic compound with multiple fused and linked aromatic rings. It contains a quinazoline-like core connected via a secondary amine to a benzimidazole-type ring, along with a substituted phenyl ring bearing an ether side chain that terminates in a secondary amide. The structure includes several nitrogen heterocycles, aromatic systems, and an isopropyl-substituted amide. Among the different synthetic routes for belumosudil, herein is presented one efficient procedure taken from a patent (Figure 10b) [58]. Belumosudil (**16**) is constructed through selective peptide coupling. The carboxylic acid intermediate **INT12** is activated using the phosphonium reagent PyBOP and the non-nucleophilic base DIPEA, which facilitates a smooth nucleophilic substitution by isopropylamine to afford the target drug in a 61% yield.

#### 2.1.8. Finerenone

**Figure 11:**
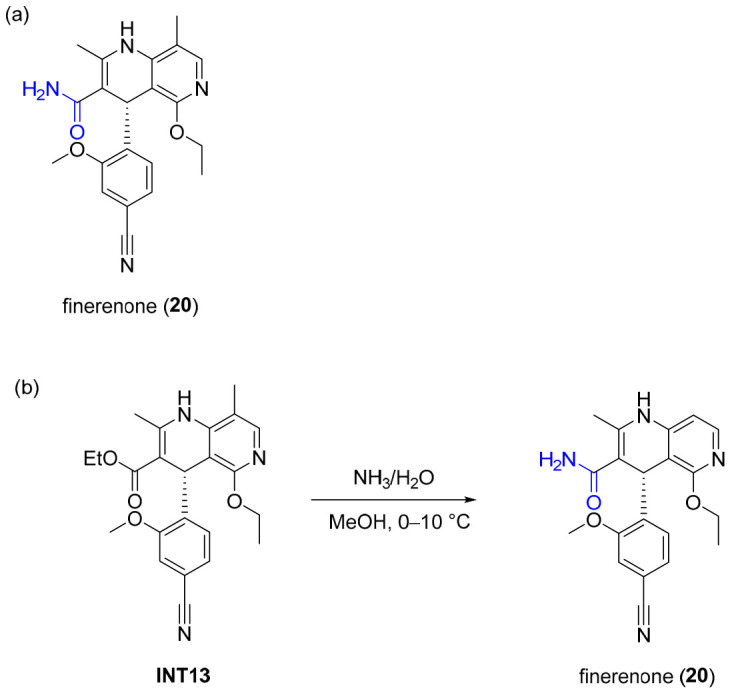
(**a**) Chemical structure of finerenone. (**b**) Final step for the synthesis of finerenone (**20**).

Approved on 9 July 2021, finerenone (**20**, brand name Kerendia^®^, Figure 11a) is a nonsteroidal mineralocorticoid receptor antagonist used to reduce the risk of chronic kidney disease progression and cardiovascular events in adults with chronic kidney disease associated with type 2 diabetes [59,60]. It works by selectively blocking mineralocorticoid receptor-mediated inflammation and fibrosis, offering renal and cardiovascular protection with a lower risk of hormonal side effects compared to older agents [60]. The structure is a polycyclic, aromatic-rich molecule featuring a fused heterocyclic core linked to a substituted phenyl ring. It contains multiple nitrogen functionalities, including a heteroaromatic ring nitrogen, a secondary amine, an amide group, and a nitrile substituent. Oxygen-containing groups include ether linkages and a methoxy substituent, contributing to polarity and hydrogen-bonding capacity. The final step to obtain finerenone is presented in Figure 11b. The corresponding ethyl ester **INT13** undergoes aminolysis using ammonia (an aqueous solution, dissolved in an alcohol like methanol). The reaction temperature in this step is 0 °C to room temperature, preferably 0 °C to 10 °C, and the reaction time is 10 to 20 h, preferably 16 to 20 h [61].

#### 2.1.9. Samidorphan

**Figure 12:**
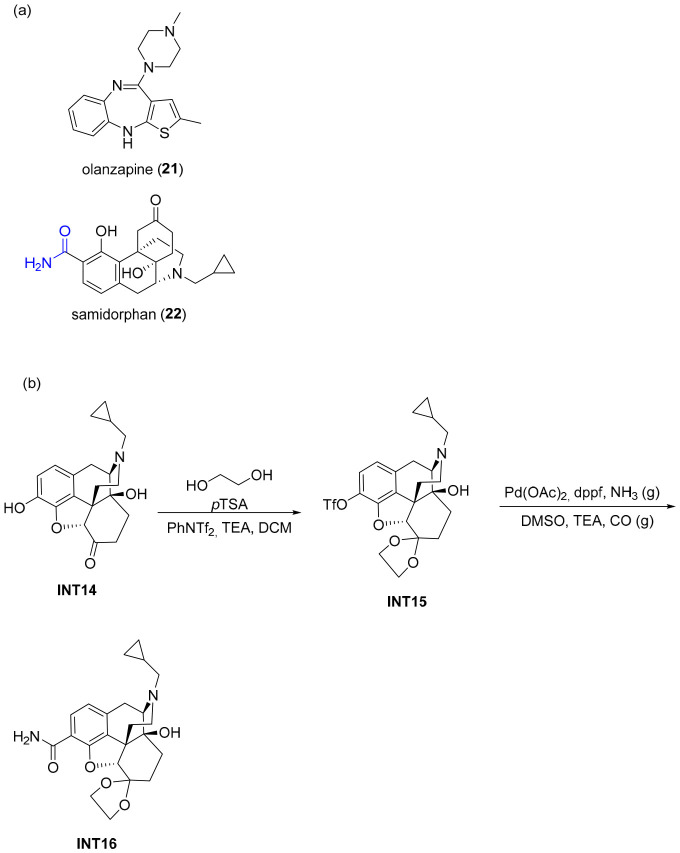
(**a**) Chemical structure of olanzapine and samidorphan. (**b**) Preparation of intermediate containing amide **INT16**.

Approved on 28 May 2021, Lybalvi^®^ (Figure 12a), a fixed-dose combination of olanzapine (**21**) and samidorphan (**22**), was indicated for the treatment of schizophrenia and bipolar I disorder. Samidorphan is included to mitigate olanzapine-associated weight gain while preserving the antipsychotic efficacy of olanzapine, improving the overall metabolic safety profile [62,63]. Only samidorphan contains an amide moiety. The preparation of the intermediate containing an amide (Figure 12b) in samidorphan starts with protection of the ketone (**INT14**) with ethylene glycol and p-TSA to form a cyclic ketal protecting group, preventing it from interfering with later steps. Next, the activation of the phenol on the aromatic ring is reacted with PhNTf_2_ and TEA to convert it into a triflate, turning a poor leaving group into an excellent one (**INT15**). Eventually, **INT15** undergoes palladium-catalyzed cross-coupling using Pd(OAc)_2_ and 1,1′-Bis(diphenylphosphino)ferrocene (dppf). In the presence of carbon monoxide (gaseous CO) and ammonia, the triflate is replaced by a primary carboxamide group to yield the final product (**INT16**) [64].

#### 2.1.10. Sotorasib

**Figure 13:**
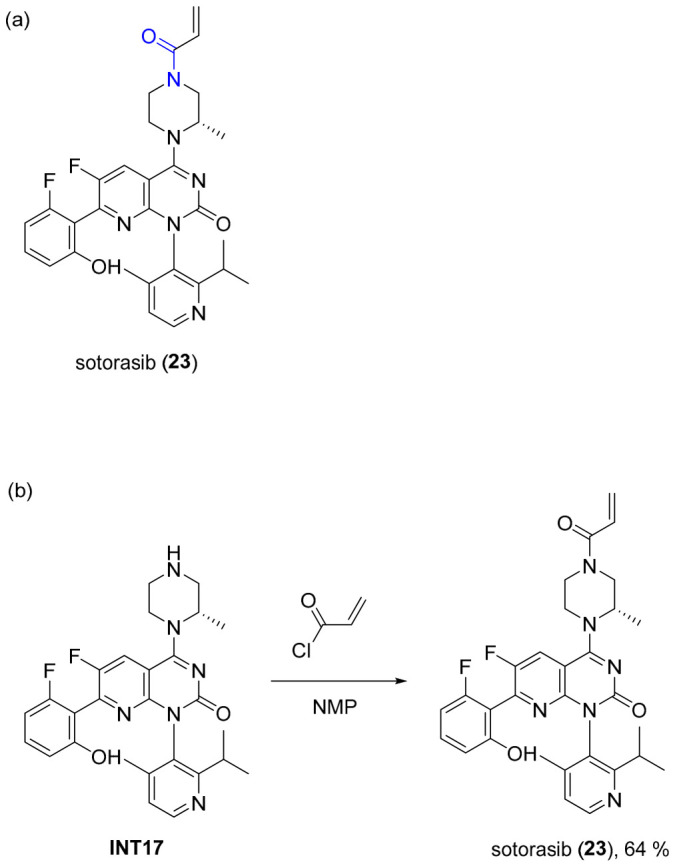
(**a**) Chemical structure of sotorasib. (**b**) Final stage preparation of sotorasib.

Approved on 28 May 2021, sotorasib (**23**, brand name Lumakras^®^, Figure 13a) is the first targeted therapy for patients with a KRAS mutation, specifically the G12C variant, which was previously considered an “undruggable” target in oncology [65]. Sotorasib works by irreversibly and selectively binding to the inactive GDP-bound state of the mutant KRAS G12C protein, effectively locking it into an inactive form to block downstream signaling pathways that drive tumor growth [66]. The acrylamide group of sotorasib is an electrophilic warhead that can freely react with nucleophiles such as thiol and amine groups [67]. The installation of an acrylic group on an amine (**INT17**) was accomplished via acryloyl chloride in NMP (Figure 13b) [68].

#### 2.1.11. Piflufolastat F 18

**Figure 14:**
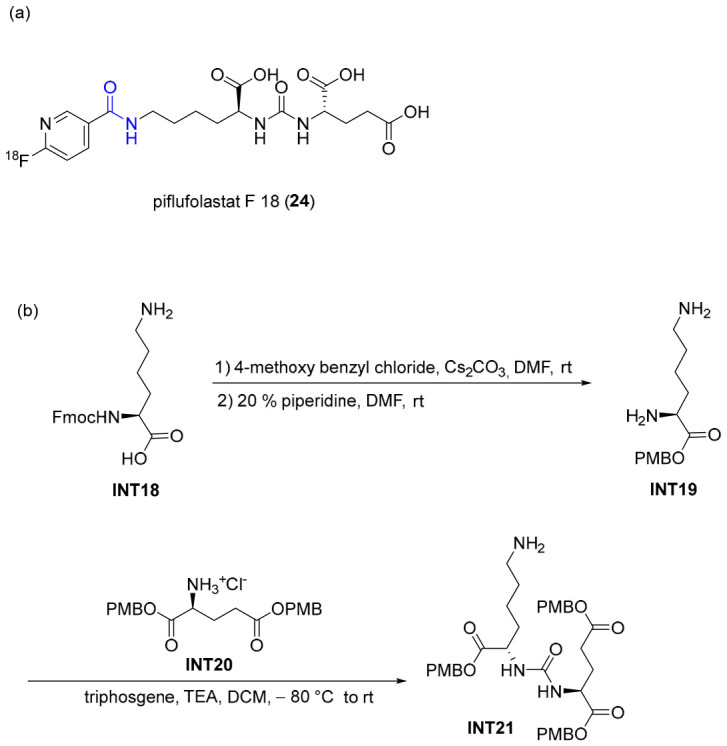
(**a**) Chemical structure of piflufolastat F 18. (**b**) Preparation of urea INT21 used in the synthesis of piflufolastat F 18.

Approved on 27 May 2021, piflufolastat F 18 (**24**, brand name Pylarify^®^, Figure 14a) is a radioactive diagnostic agent used in PET/CT imaging to detect prostate-specific membrane antigen (PSMA)-positive lesions in men with prostate cancer. It is specifically for identifying suspected metastasis before initial treatment or detecting recurrent cancer based on elevated PSA levels [69,70]. Its chemistry revolves around a highly specific urea-based scaffold designed to inhibit the prostate-specific membrane antigen (PSMA) [71]. The synthesis (Figure 14b) originates from Nε-Boc-Nα-Fmoc-l-lysine (**INT18**), which first protects acidic functionality with *p*-methoxy benzyl chloride, and secondly, Fmoc is cleaved by using a 20% piperidine solution to afford the derivative **INT19**. Urea **INT21** was obtained by treating bis-4-methoxybenzyl-l-glutamate·HCl (**INT20**) with triphosgene and triethylamine at −80 °C, followed by in situ trapping of the isocyanate intermediate by the addition of **INT20** [72].

#### 2.1.12. Serdexmethylphenidate

**Figure 15:**
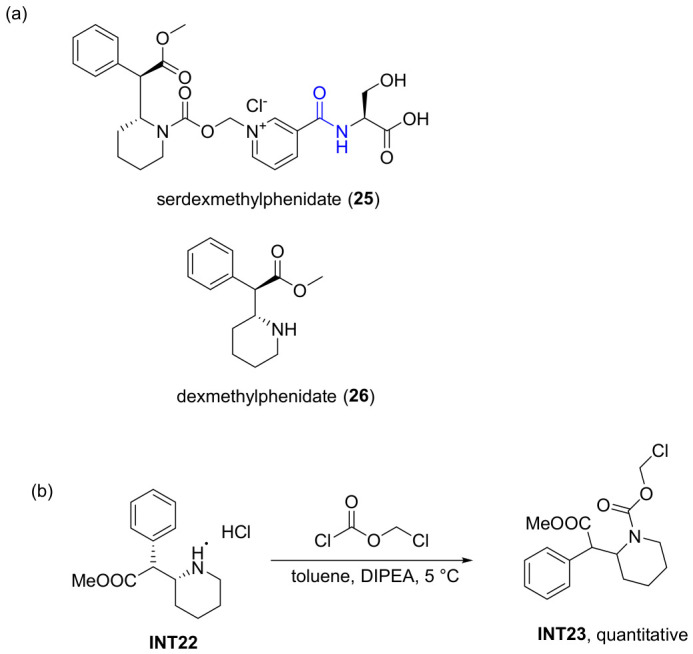
(**a**) Chemical structure of serdexmethylphenidate and dexmethylphenidate. (**b**) Preparation of intermediate INT23, useful for the preparation of serdexmethylphenidate.

Azstarys^®^ (Figure 15a, the combined formulation of serdexmethylphenidate **25** and dexmethylphenidate **26**) was approved by the U.S. Food and Drug Administration (FDA) in March 2021 for medical use in the United States for the once-daily treatment of Attention-Deficit/Hyperactivity Disorder (ADHD) in patients aged 6 years and older [73,74]. Azstarys^®^ is a central nervous system (CNS) stimulant that pairs immediate-release dexmethylphenidate with serdexmethylphenidate, a prodrug that gradually converts to dexmethylphenidate after ingestion, aiming to provide a rapid onset and extended duration of symptom control throughout the day [75]. The released dexmethylphenidate then inhibits the reuptake of dopamine and norepinephrine by blocking the dopamine transporter (DAT) and norepinephrine transporter (NET) in the central nervous system, increasing the availability of these neurotransmitters in synaptic clefts—particularly in the prefrontal cortex [76]. Serdexmethylphenidate contains a hydrolyzable ester bond that links the dexmethylphenidate core to its promoiety, which is essential for its function as a prodrug. This ester bond (specifically a carbamate ester) is gradually cleaved by enzymatic hydrolysis after oral administration, releasing the active dexmethylphenidate. Synthetically, reaction of methyl (R)-2-phenyl-2-((R)-piperidin-2-yl)acetate hydrochloride **INT22** with chloromethyl chloroformate using DIPEA as a base generated the carbamate **INT23** in quantitative yield (Figure 15b) [32].

#### 2.1.13. Melphalan Flufenamide

**Figure 16:**
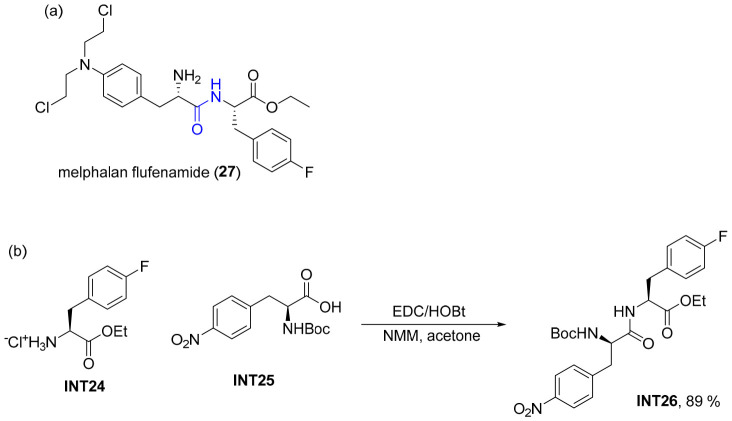
(**a**) Chemical structure of melphalan flufenamide. (**b**) Preparation of intermediate **INT26**, useful for the preparation of melphalan flufenamide.

Approved on 26 February 2021, melphalan flufenamide (**27**, brand name Pepaxto^®^, Figure 16a) is a peptide-based drug designed to deliver the alkylating agent melphalan (**28**) preferentially into cancer cells with high aminopeptidase activity [77]. It received accelerated FDA approval in combination with dexamethasone for adults with heavily pretreated relapsed or refractory multiple myeloma based on response rates from the phase 2 HORIZON trial [78]. However, subsequent confirmatory data did not demonstrate a favorable benefit–risk profile, and the FDA withdrew its approval in February 2024 [79].

Melphalan flufenamide is designed as a prodrug. Melphalan itself is polar and enters cells relatively inefficiently. By linking melphalan to a short peptide via an amide bond, the molecule becomes more lipophilic, allowing it to cross cell membranes much more easily [80]. Melphalan flufenamide is a neutral, relatively lipophilic prodrug with aromatic and amide/ester functionalities, which makes it preferentially soluble in polar aprotic solvents. The synthesis of synthon (**INT26**), important for the synthesis of melphalan flufenamide, is accomplished via condensation (EDC/HOBt) between amine (**INT24**) and (S)-2-((tert-butoxycarbonyl)amino)-3-(4-nitrophenyl)propanoic acid (**INT25**) in a good yield (89%, Figure 16b) [32].

#### 2.1.14. Fosdenopterin

**Figure 17:**
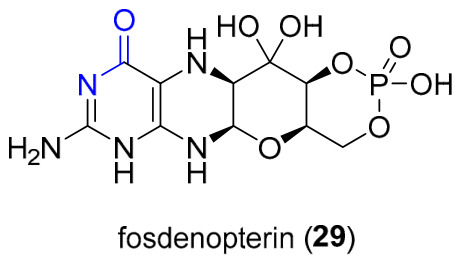
Chemical structure of fosdenopterin.

Approved on 26 February 2021, fosdenopterin (**29**, brand name Nulibry^®^, Figure 17) is a medicine that has been studied largely for its ability to treat molybdenum cofactor deficiency (MCD), a rare metabolic condition. This disorder is caused by a deficiency in the creation of molybdenum cofactor, which is required for the proper functioning of several essential enzymes [81]. Chemically, fosdenopterin has a carbonyl within the pterin heterocycle (a pteridin-4-one), but this is part of an aromatic heterocyclic ring system (lactam-like), not a discrete –C(=O)–NH– amide linkage. The amide linkage was already established in the primary building blocks, shifting the synthetic focus toward peripheral modifications.

#### 2.1.15. Trilaciclib

**Figure 18:**
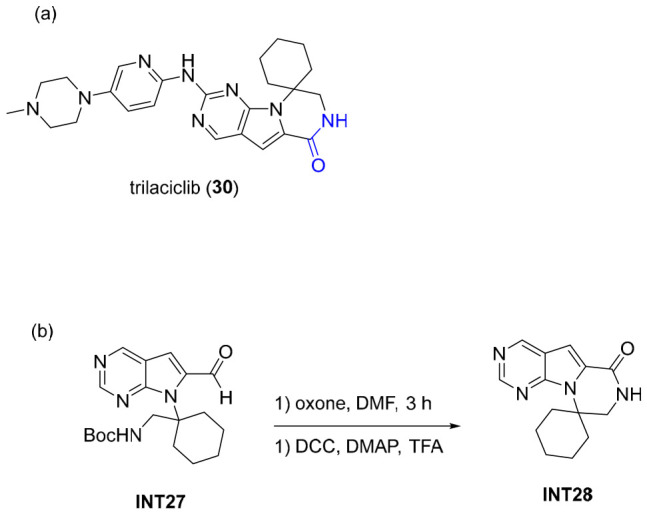
(**a**) Chemical structure of trilaciclib. (**b**) Formation of lactam in trilaciclib.

Approved on 12 February 2021, trilaciclib (**30**, brand name Cosela^®^, Figure 18a) is a small-molecule cyclin-dependent kinase (CDK) 4/6 inhibitor developed by G1 Therapeutics that is used to decrease the incidence of chemotherapy-induced myelosuppression in adult patients receiving certain chemotherapeutic regimens for extensive-stage small cell lung cancer (ES-SCLC) by transiently arresting hematopoietic stem and progenitor cells in G1 phase, protecting bone marrow from damage [82,83]. The key synthetic step to form the lactam is presented in Figure 18b. First, the formyl group on **INT27** is oxidized to a carboxylic acid using Oxone in DMF. Following this, trifluoroacetic acid (TFA) cleaves the *tert*-butyloxycarbonyl (Boc) protecting group to liberate the primary amine. Finally, the coupling reagent DCC and catalyst DMAP facilitate an intramolecular amide bond formation between the newly generated carboxylic acid and the free amine. This lactamization sequence successfully closes the seven-membered ring, yielding the spirocyclic architecture seen in **INT28** [84].

#### 2.1.16. Tepotinib

**Figure 19:**
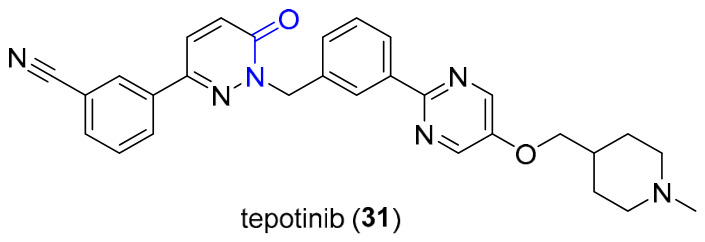
Chemical structure of tepotinib.

Approved on 3 February 2021, tepotinib (**31**, brand name Tepmetko^®^, Figure 19) is a targeted oral therapy designed for adults with advanced or metastatic non-small-cell lung cancer (NSCLC) that has specific MET exon 14 skipping alterations [85,86]. As a highly selective inhibitor, it works by blocking the MET protein, which is responsible for cancer cell growth, survival, and spread. It is especially useful for patients with this aggressive form of lung cancer as it provides a personalized, daily treatment option that has demonstrated meaningful clinical activity, often leading to tumor shrinkage in both newly diagnosed and previously treated patients [87,88]. Tepotinib contains a cyclic amide known as a pyridazinone (specifically 6-oxo-1,6-dihydropyridazin-3-yl), which is a 6-membered lactam ring. The pyridazinone-containing building block was introduced intact, rather than being generated de novo during the synthesis [80].

#### 2.1.17. Voclosporin

**Figure 20:**
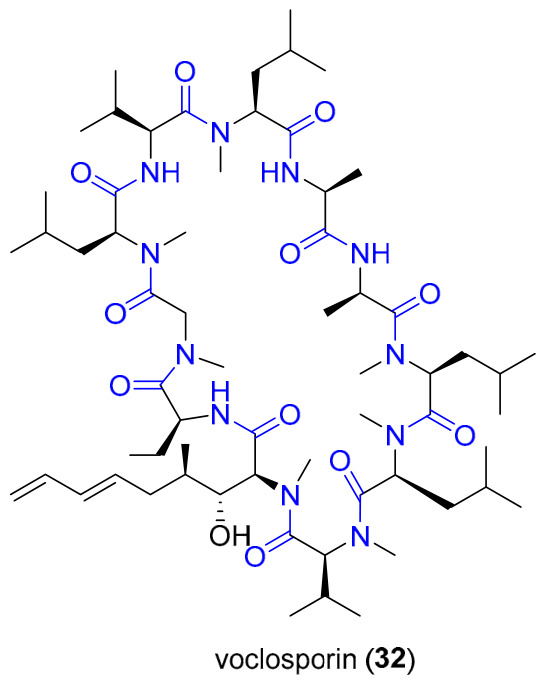
Chemical structure of **voclosporin**.

Approved by the FDA on 22 January 2021, voclosporin (**32**, brand name Lupkynis^®^, Figure 20) received its first global approval for the treatment of adult patients with active lupus nephritis (LN) in combination with a background immunosuppressive therapy, typically mycophenolate mofetil and steroids [89].

Voclosporin is a novel oral calcineurin inhibitor that suppresses T-cell activation and stabilizes podocytes, thereby reducing proteinuria in lupus nephritis [90]. It is a cyclic undecapeptide (11 amino acids), produced by non-ribosomal peptide synthesis, like cyclosporine A [91]. Most residues are non-polar or unusual amino acids, which enhances membrane permeability and calcineurin binding [91]. In summary, voclosporin is a potent cyclosporine derivative, and is safer, more efficacious, and more tolerable than other calcineurin inhibitors [92]. Voclosporin is synthesized using intermediate cyclosporin A as raw material by chemical reaction [93].

#### 2.1.18. Cabotegravir

**Figure 21:**
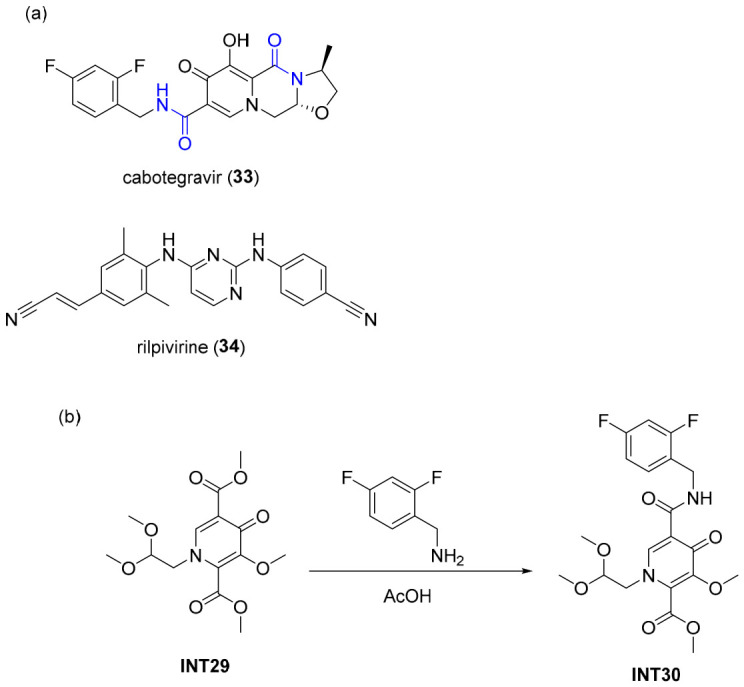
(**a**) Chemical structure of cabotegravir and rilpivirine. (**b**) Preparation of **INT30**, used in the synthesis of rilpivirine.

Approved on 21 January 2021, Cabenuva^®^ (Figure 21a) is a combination of cabotegravir **33** plus rilpivirine **34**; it is the first long-acting injectable complete regimen for HIV-1 treatment [94], given monthly or every two months for virologically suppressed adults, combining the integrase inhibitor cabotegravir with the non-nucleoside reverse transcriptase inhibitor (NNRTI) rilpivirine to replace daily oral therapy [95]. Cabotegravir has an amide in the form of a lactam and a classic amide that connect to the halobenzyl moiety. This latter amide carbonyl next to the halobenzyl linker is devoid of metal chelation (magnesium ions at the pharmacophore). This increases the linker’s torsional flexibility, allowing it to better react to changed mutant surroundings [96]. The preparation of the secondary amide linkage is shown in Figure 21b. This reaction shows a regioselective nucleophilic acyl substitution (amidation) converting **INT29** to **INT30**. The starting material **INT29** contains two distinct methyl ester groups on its pyridin-4-one core: one at the C-3 position and one at the C-5 position. The primary amine, 2,4-difluorobenzylamine, selectively attacks the more electrophilic C-3 methyl ester rather than the C-5 ester. This selectivity occurs because the ester at C-3 is directly conjugated with the electron-withdrawing carbonyl group of the pyridin-4-one ring, making its carbonyl carbon highly susceptible to nucleophilic attack. Under the acidic catalysis of acetic acid, the amine displaces the methoxy leaving group to form the corresponding secondary amide, yielding **INT30** with the C-5 methyl ester left completely intact [97].

#### 2.1.19. Difelikefalin

**Figure 22:**
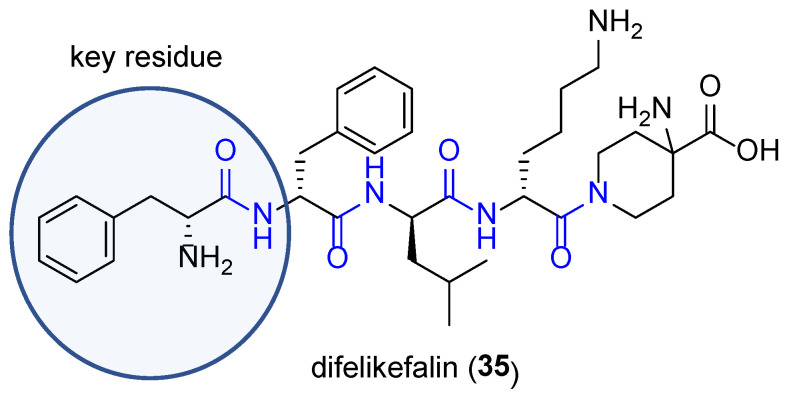
Chemical structure of difelikefalin.

Approved by the FDA in August 2021, difelikefalin (**35**, brand name Korsuva^®^/Kapruvia^®^, Figure 22) is a first-in-class, peripherally restricted kappa opioid receptor agonist (KORA) approved to treat moderate-to-severe itching (pruritus) in adults with chronic kidney disease (CKD) on hemodialysis [98,99]. Difelikefalin is a synthetic peptide made from D-amino acids, specifically comprising D-phenylalanine (D-Phe), D-leucine (D-Leu), and D-lysine (D-Lys), along with the unnatural amino acid 4-aminopiperidine-4-carboxylic acid. The *N*-terminal D-phenylamine (Phe) functional group (Figure 22) is a key pharmacophore, and this area is very sensitive to the binding activity of KORA [100]. The peptide backbone of the drug was efficiently assembled using EDC as the primary coupling reagent, facilitating the sequential joining of the amino acid building blocks in near-quantitative yields [101].

### 2.2. FDA-Approved Drugs in 2022 Containing Amide Group

In 2022, the FDA approved a total of 37 drugs, of which 22 were new chemical entities [28]. Compared with 2021, 2022 saw a decline in the number of approved chemical entities, which may partly reflect ongoing disruptions related to the COVID-19 pandemic.

While the FDA did not give a specific cause for the decline in approvals, the agency was a regular focus of criticism in 2022 for potentially moving too fast on some pharmaceuticals, especially those in the accelerated approval process [102]. The reduced number of approved drugs is correlated with a small number of small-molecule amides.

#### 2.2.1. Futibatinib

**Figure 23:**
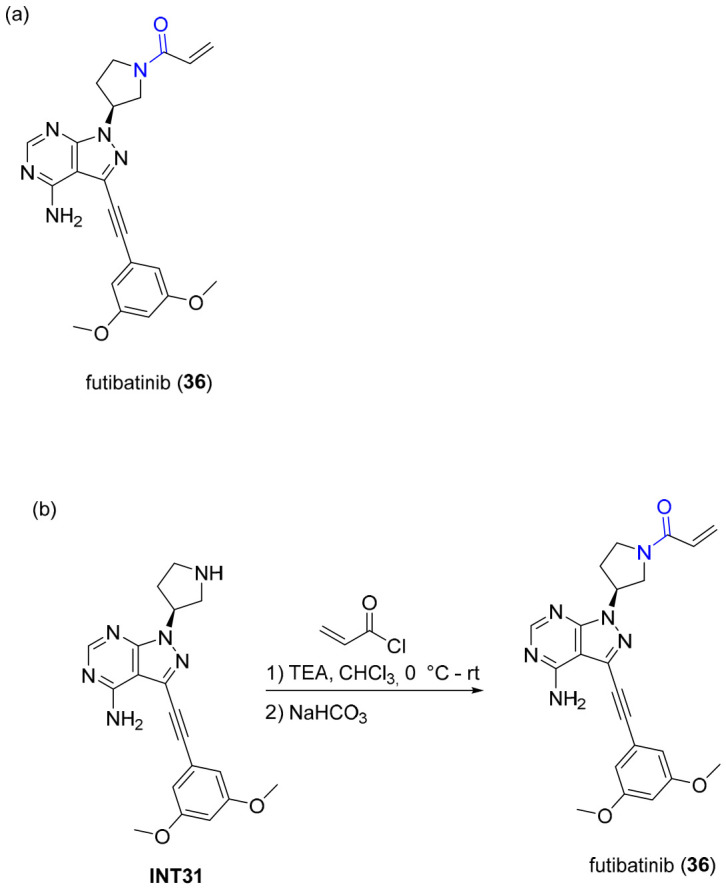
(**a**) Chemical structure of futibatinib. (**b**) Last step preparation of futibatinib.

Approved on 30 September 2022, futibatinib (**36**, brand name Lytgobi^®^, Figure 23a) is a selective, irreversible fibroblast growth factor receptor (FGFR) inhibitor used for the treatment of adults with previously treated, unresectable locally advanced or metastatic cholangiocarcinoma harboring FGFR2 gene fusions or rearrangements [103]. The 3,5-dimethoxybenzene structure resides in the back pocket of FGFR1, whereas the acrylamide structure forms a covalent bond with cysteine 488 [104]. The secondary amine of the pyrrolidine ring in **INT31** acts as a nucleophile, selectively attacking the carbonyl carbon of acryloyl chloride via a nucleophilic acyl substitution. Triethylamine is employed as a non-nucleophilic base in chloroform to scavenge the generated hydrochloric acid byproduct and prevent protonation of the remaining starting material. The reaction selectively functionalizes the aliphatic secondary amine while leaving the less nucleophilic exocyclic aromatic amine untouched. A subsequent aqueous sodium bicarbonate wash neutralizes residual salts, cleanly furnishing the target acrylamide warhead (Figure 23b) [105].

#### 2.2.2. Olutasidenib

**Figure 24:**
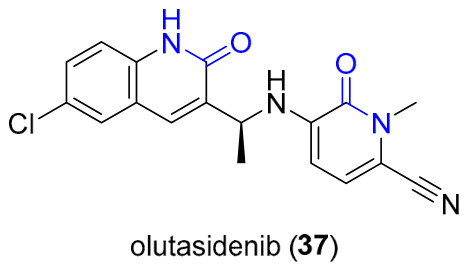
Chemical structure of olutasidenib.

Approved on 1 December 2022, olutasidenib (**37**, brand name Rezlidhia^®^, Figure 24) is an oral, small-molecule for the treatment of adult patients with relapsed or refractory acute myeloid leukemia (AML) harboring a susceptible isocitrate dehydrogenase-1 (IDH1) mutation. It reduces the production of the oncometabolite 2-hydroxyglutarate and thereby promotes differentiation of leukemic blasts [106].

Its structure–activity relationship (SAR) is centered on a quinoline-2-one scaffold that fits into an allosteric pocket, allowing it to specifically target mutant forms while sparing wild-type IDH1 [107,108]. The 2-pyridone-containing building block was introduced intact rather than being generated de novo during the synthesis [109].

#### 2.2.3. Adagrasib

**Figure 25:**
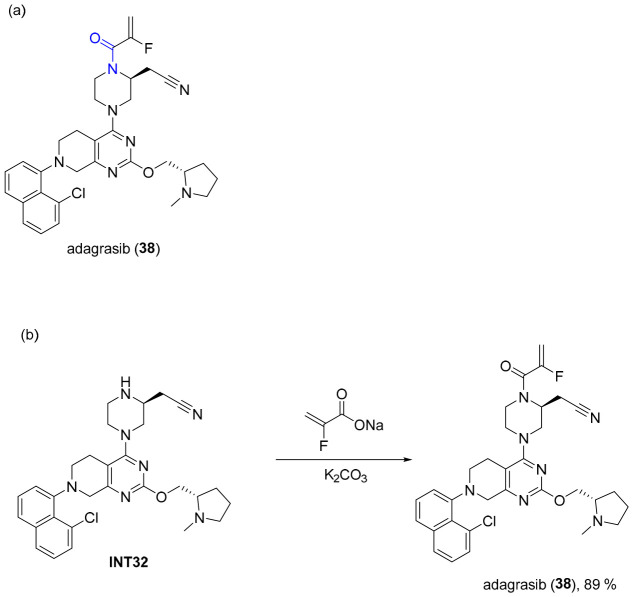
(**a**) Chemical structure of adagrasib. (**b**) Final step preparation of adagrasib.

Approved on 12 December 2022, adagrasib (**38**, brand name Krazati^®^, Figure 25a) is a small molecule for the treatment of adult patients with locally advanced or metastatic nonsmall cell lung cancer (NSCLC harboring a KRAS G12C mutation who have received at least one prior systemic therapy [110]. Adagrasib is an orally bioavailable, covalent small-molecule inhibitor that selectively targets the KRAS G12C mutant protein by irreversibly binding to the cysteine residue at position 12, locking KRAS in its inactive GDP-bound state and thereby inhibiting downstream oncogenic signaling through pathways such as MAPK and PI3K [111]. The secondary amine on the piperazine ring of intermediate (**INT32**) acts as a nucleophile, attacking the activated carbonyl center of sodium 2-fluoroacrylate. Potassium carbonate serves as a mild base to facilitate the coupling reaction. This nucleophilic substitution cleanly installs the electrophilic 2-fluoroacrylamide warhead onto the piperazine nitrogen while leaving the other tertiary amine centers intact [112].

#### 2.2.4. Deucravacitinib

**Figure 26:**
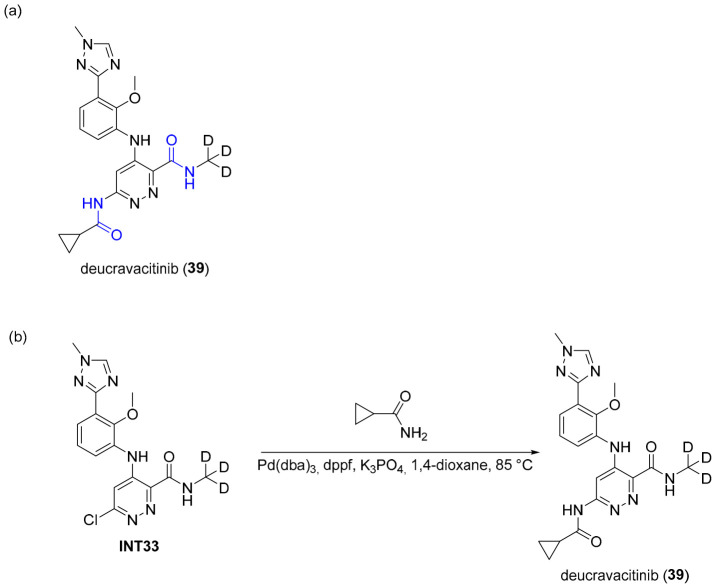
(**a**) Chemical structure of deucravacitinib. (**b**) Final step preparation of deucravacitinib.

Approved on 9 September 2022, deucravacitinib (**39**, brand name: Sotyktu^®^, Figure 26a) is a first-in-class, oral medication that represents a major leap forward in treating autoimmune diseases [113]. It is a first-in-class, oral, selective allosteric inhibitor of tyrosine kinase 2 (TYK2), a member of the JAK family, approved for moderate-to-severe plaque psoriasis [114]. The inclusion of deuterium strengthens chemical bonds, which reduces metabolic degradation and potentially allows for lower or less frequent dosing while maintaining efficacy [115,116]. The cyclopropyl amide in deucravacitinib is a masterclass in molecular engineering. It is a multifunctional group that is essential for the drug’s high potency and exceptional selectivity to limit hERG liability [117].

The final step in the preparation of deucravacitinib is shown in Figure 26b. The reaction cross-couples the primary cyclopropanecarboxamide reagent with the electrophilic C-6 carbon of the pyridazine core (**INT33**), displacing the chlorine atom. This C–N bond formation is driven by a catalytic system comprising tris(dibenzylideneacetone)dipalladium(0) and the bidentate phosphine ligand dppf, which facilitate oxidative addition and reductive elimination cycles. Potassium phosphate acts as the base to deprotonate the amide in 1,4-dioxane at 85 °C, efficiently introducing the cyclopropyl amide group without disturbing the existing deuterated amide framework [105].

#### 2.2.5. Mitapivat

**Figure 27:**
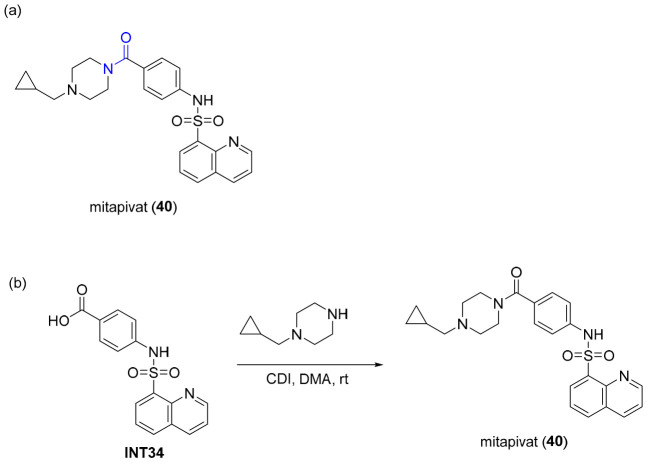
(**a**) Chemical structure of mitapivat. (**b**) Final step preparation of mitapivat.

Approved on 17 February 2022, mitapivat (**40**, brand names Pyrukynd^®^ and Aqvesme^®^, Figure 27a) is a first-in-class oral pyruvate kinase activator that treats hemolytic anemia in adults with pyruvate kinase (PK) deficiency. It works by increasing PK activity to boost red blood cell health. It is also approved for treating anemia in adults with alpha- or beta-thalassemia [118]. It features a quinoline-8-sulfonamide core, a central phenyl ring, and a cyclopropylmethylpiperazine moiety. This reaction outlines a CDI-mediated amide coupling to synthesize mitapivat from carboxylic acid. 1,1′-Carbonyldiimidazole (CDI) first activates the carboxylic acid group of intermediate (**INT34**) by converting it into a reactive acyl imidazole intermediate. The free secondary amine of the piperazine dihydrochloride salt then undergoes nucleophilic acyl substitution at this activated carbonyl center. Operating in *N*,*N*-dimethylacetamide (DMA) at room temperature for 4 h, this protocol cleanly couples the fragments to generate the central amide linkage of the drug while leaving the sulfonamide and quinoline functionalities completely unaffected (Figure 27b) [105].

#### 2.2.6. Lutetium Lu 177 Vipivotide Tetraxetan

**Figure 28:**
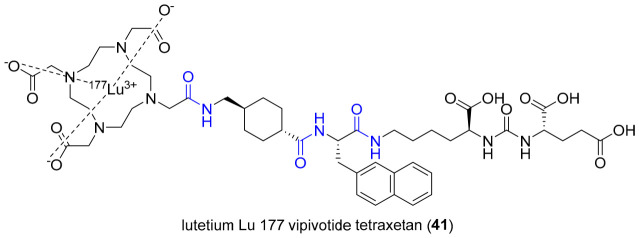
Chemical structure of lutetium Lu 177 vipivotide tetraxetan.

Approved on 23 March 2022, lutetium Lu 177 vipivotide tetraxetan (**41**, brand name Pluvicto^®^, Figure 28) is an FDA-approved, targeted radioligand therapy for adult patients with advanced, PSMA-positive metastatic castration-resistant prostate cancer (mCRPC) previously treated with chemotherapy and hormone therapy. It works by delivering radiation directly to cancer cells, significantly extending survival and reducing tumor size, with common side effects including fatigue, dry mouth, and nausea. Its structure is composed of PSMA-617, a human prostate-specific membrane antigen (PSMA)-targeting ligand, conjugated to the beta-emitting radioisotope lutetium, ^177^Lu [119]. The synthesis of lutetium Lu 177 vipivotide tetraxetan is achieved via multi-step coupling mediated by O-(Benzotriazol-1-yl)-N,N,N’,N’-bis (tetramethylene)uronium Hexafluorophosphate (HBTU) and DIPEA as a base [105].

#### 2.2.7. Terlipressin

**Figure 29:**
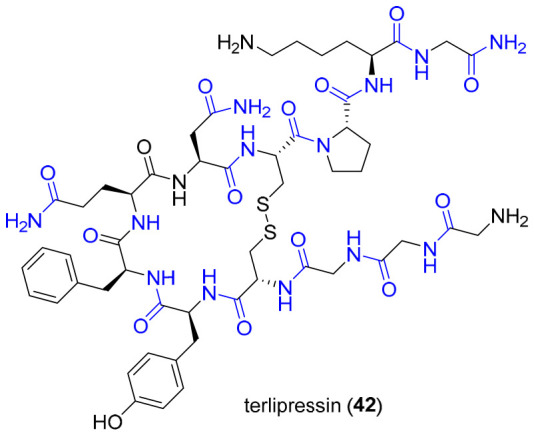
Chemical structure of terlipressin.

Approved on 14 September 2022, terlipressin (**42**, brand name Terlivaz^®^, Figure 29) was approved by the U.S. Food and Drug Administration for improving kidney function in adults with hepatorenal syndrome (HRS) with rapid reduction in kidney function, a life-threatening complication of advanced liver disease [120]. It is an analog of vasopressin, and it acts by causing vasoconstriction of the splanchnic vasculature [121]. Its core structure consists of a nine-amino acid peptide chain that is cyclized by a disulfide bond between two cysteine residues, a hallmark feature shared with vasopressin analogs [122]. The synthesis of terlipressin is achieved via multi-step coupling mediated by *N*-Hydroxy-5-norbornene-2,3-dicarboximide (HONB) and DCC [123].

### 2.3. FDA-Approved Drugs in 2023 Containing Amide Group

In 2023, the FDA approved a total of 55 drugs, of which 38 were new chemical entities [28]. As such, 2023 was a very good year for drug discovery, especially for small-molecule innovation and clinically meaningful advances. It reflects a mature, productive ecosystem rather than a sudden breakthrough era, but it is a clear signal that modern drug discovery is delivering at scale.

#### 2.3.1. Nirogacestat

**Figure 30:**
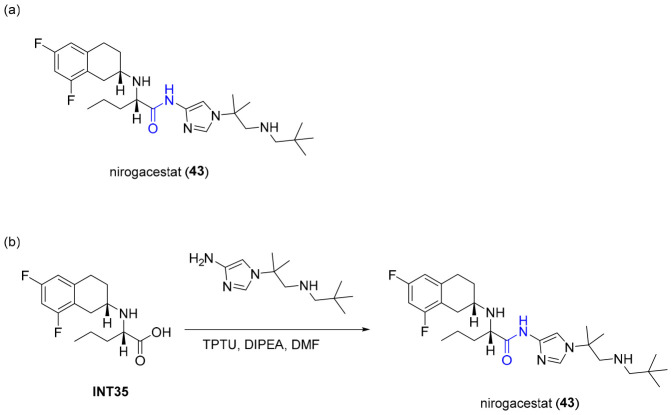
(**a**) Chemical structure of nirogacestat. (**b**) Final step preparation of nirogacestat.

Approved on 27 November 2023, nirogacestat (**43**, brand name Ogsiveo^®^, Figure 30a) is an orally available, small-molecule gamma-secretase inhibitor developed for the treatment of adults with progressing desmoid tumors, a rare and locally aggressive soft-tissue neoplasm. By inhibiting gamma-secretase, nirogacestat suppresses Notch signaling, a key pathway implicated in the growth and survival of desmoid tumor cells [124,125]. FDA approval was based on the phase 3 DeFi trial, which demonstrated significant improvements in progression-free survival, tumor response, and patient-reported outcomes compared with placebo, establishing nirogacestat as the first approved systemic therapy specifically for this disease [125,126]. Chemically, it is an imidazole-based drug containing an amide group. The final step for the synthesis of nirogacestat is performed under 2-(2-Pyridon-1-yl)-1,1,3,3-tetramethyluronium tetrafluoroborate (TPTU) and DIPEA as a base, with the carboxylic acid (**INT35**) as starting material (Figure 30b) [127].

#### 2.3.2. Capivasertib

**Figure 31:**
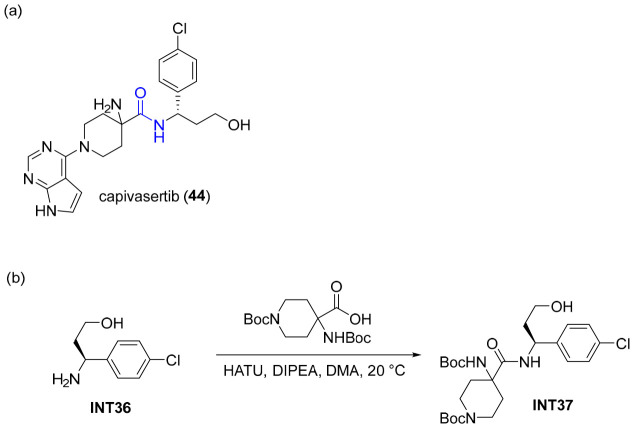
(**a**) Chemical structure of capivasertib. (**b**) Preparation of **INT37**, used in the synthesis of capivasertib.

Approved by the U.S. FDA on 16 November 2023, capivasertib (**44**, brand name Truqap^®^, Figure 31a) is a small molecule that, in combination with fulvestrant, has an indication for adult patients with advanced or metastatic hormone receptor (HR)-positive, HER2-negative breast cancer that has specific genetic alterations (PIK3CA, AKT1, or PTEN) [128]. Capivasertib can effectively inhibit the AKT pathway, leading to dephosphorylation of key downstream targets [129]. Fulvestrant serves as the foundational hormonal therapy designed to stop hormone receptor-positive breast cancer cells from growing by inhibiting estrogen action [130]. The formation of the amide bond in capivasertib is shown in Figure 31b. The transformation relies on HATU as the coupling reagent to convert the carboxylic acid (**INT36**) into a highly reactive activated ester that ultimately is converted to amide (**INT37**). DIPEA serves as the non-nucleophilic base to scavenge acid, while DMA (dimethylacetamide) provides a polar, aprotic solvent environment to keep all components dissolved at 20 °C [129].

#### 2.3.3. Repotrectinib

**Figure 32:**
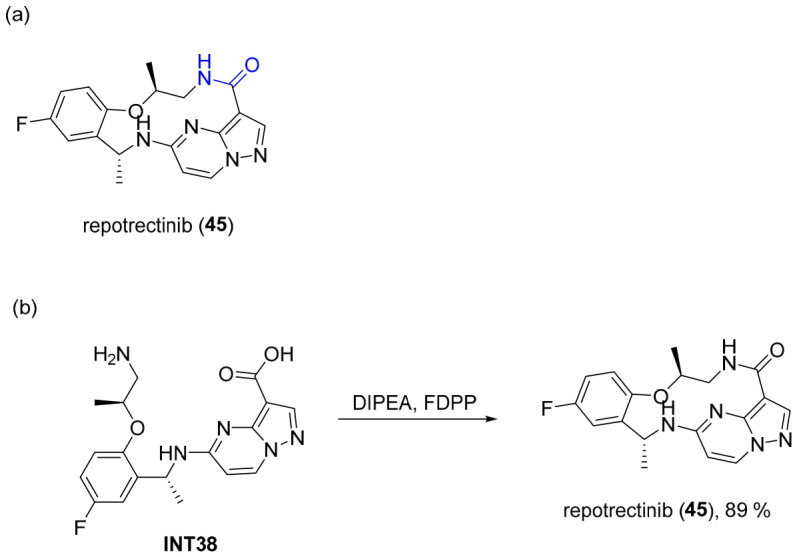
(**a**) Chemical structure of repotrectinib. (**b**) Final step preparation of repotrectinib.

Approved on 15 November 2023, repotrectinib (**45**, brand name Augtyro^®^, Figure 32a) is a next-generation tyrosine kinase inhibitor (TKI) approved by the FDA for treating specific types of advanced cancer driven by genetic alterations [131]; traditional approval was granted for adult patients with locally advanced or metastatic ROS1-positive non-small-cell lung cancer (NSCLC). This was the first approval to specifically include patients previously treated with a ROS1 TKI [132]. The first-generation TRK inhibitors’ acquired resistance can be addressed by using the macrocyclization approach, which makes the inhibitor’s molecular structure more compact [133]. Repotrectinib is obtained via using pentafluorophenyl diphenylphosphinate (FDPP) as peptide coupling starting from intermediate (**INT38**) [134].

#### 2.3.4. Fruquintinib

**Figure 33:**
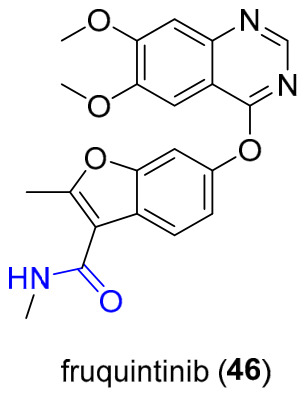
Chemical structure of fruquintinib.

Approved on 8 November 2023, fruquintinib (**46**, brand name Fruzaqla^®^, Figure 33) is an oral, selective inhibitor of vascular endothelial growth factor receptors for the treatment of adult patients with metastatic colorectal cancer (mCRC) who have previously received fluoropyrimidine-, oxaliplatin-, and irinotecan-based chemotherapy, an anti-VEGF therapy, and, if RAS wild-type and medically appropriate, an anti-EGFR therapy [135,136]. Its main pharmacological use is in refractory metastatic colorectal cancer, where it improves survival compared with placebo by inhibiting the VEGF pathway that tumors use to grow new blood vessels [137]. Beyond its approved CRC use, fruquintinib continues to be studied in other cancer indications [138,139]. The H-bond-rich region was engaged by an amide group forming H-bonds with specific amino acids (glutamic acid and aspartic acid) in the binding pocket [140]. The secondary amide building block was introduced intact rather than being generated de novo during the synthesis [141].

#### 2.3.5. Momelotinib

**Figure 34:**
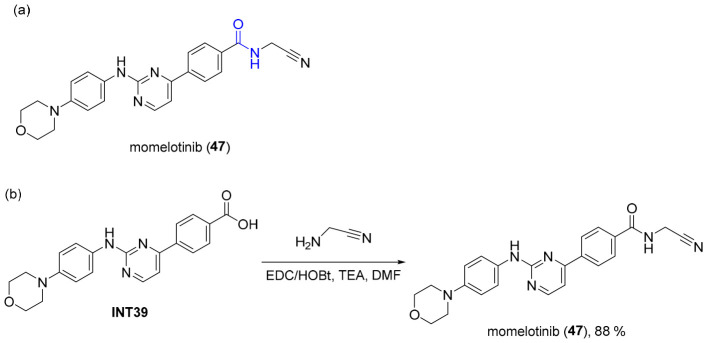
(**a**) Chemical structure of momelotinib. (**b**) Final step preparation of momelotinib.

Approved on 15 September 2023, momelotinib (**47**, brand name Ojjaara^®^, Figure 34a) is an oral kinase inhibitor targeting Janus kinase 1/2 (JAK1), JAK2 for the treatment of adult patients with intermediate- or high-risk myelofibrosis who have anemia [142]. Its unique combination of activities helps reduce constitutional symptoms, address splenomegaly (enlarged spleen), and decrease hepcidin levels to alleviate anemia in people with myelofibrosis [143]. This indication fills an important gap in managing myelofibrosis with anemia, a condition marked by bone marrow dysfunction, low blood counts, fatigue, and other disease-related symptoms [144]. The last step for preparing momelotinib is shown in Figure 34b. The transformation utilizes **INT39**, aminoacetonitrile, EDC as the carbodiimide activating agent, and HOBt as a racemization-suppressing additive to generate a reactive active ester. TEA acts as the base to deprotonate the amine, and DMF serves as the polar aprotic solvent [129].

#### 2.3.6. Lotilaner

**Figure 35:**
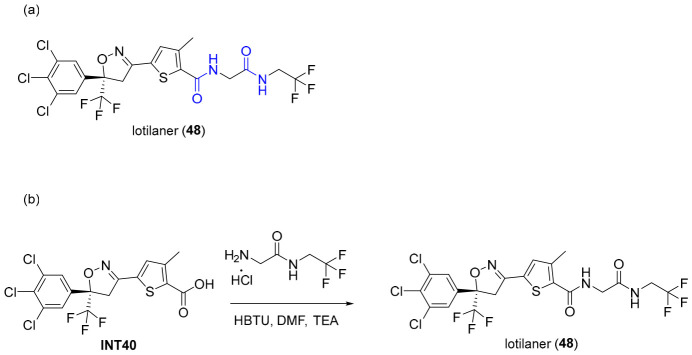
(**a**) Chemical structure of lotilaner. (**b**) Final step preparation of lotilaner.

Approved on 25 July 2023, lotilaner (**48**, brand name Xdemvy^®^, Figure 35a) is an isoxazoline-class antiparasitic drug used to kill parasites such as mites, fleas, and ticks. It is available in both human and veterinary formulations [145,146]. Lotilaner works by targeting parasite chloride channels, leading to the paralysis and death of mites, fleas, and ticks. In veterinary use, isoxazolines can occasionally cause neurologic adverse reactions, such as tremors, ataxia, or seizures, so careful dosing and veterinary supervision are recommended [147,148]. The amide bonds are introduced in lotilaner via the HBTU coupling reagent starting from intermediate **INT40** (Figure 35b). Lotilaner (**48**) is generated by efficient amide bond formation while fully preserving the halogenated groups and stereochemical configuration [127].

#### 2.3.7. Ritlecitinib

**Figure 36:**
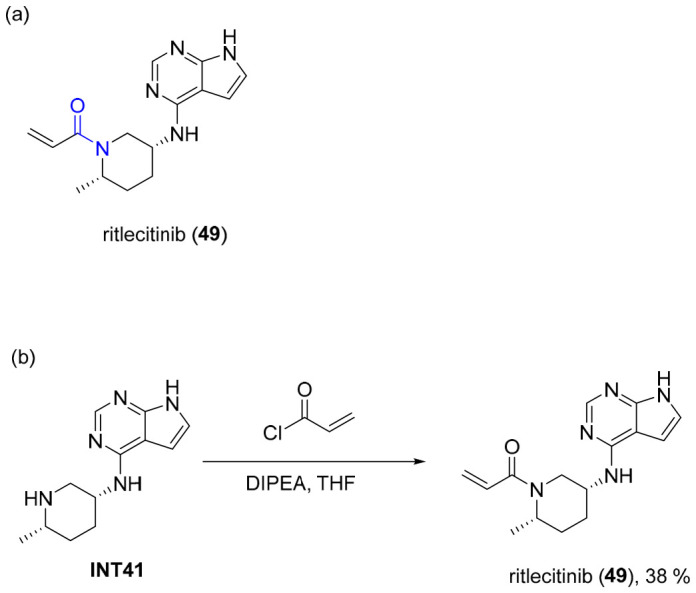
(**a**) Chemical structure of ritlecitinib. (**b**) Final step preparation of ritlecitinib.

Approved on 23 June 2023, ritlecitinib (**49**, brand name LITFULO^®^, Figure 36a) is for the treatment of severe alopecia areata in adults and adolescents aged 12 years and older [149]. It is taken orally once daily. This approval marked ritlecitinib as the first treatment specifically indicated for adolescents with this condition and the second JAK inhibitor (the first was baricitinib [150]) that was overall approved for alopecia areata in the U.S [151]. Chemically, it contains an acrylamide moiety (Figure 36b) whose installation is mediated by acryloyl chloride on **INT41** and DIPEA in THF [152].

#### 2.3.8. Nirmatrelvir and Ritonavir

**Figure 37:**
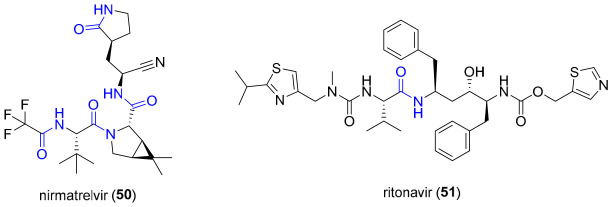
Chemical structure of nirmatrelvir and ritonavir.

Approved on 25 May 2023, the combination of nirmatrelvir **50** plus ritonavir **51** (Paxlovid^®^) was among the first oral antivirals shown to significantly reduce hospitalization and death in high-risk COVID-19 patients [153,154]. Nirmatrelvir (Figure 37) is an oral antiviral protease inhibitor that targets the SARS-CoV-2 main protease (Mpro, also called 3CLpro) [155]. By inhibiting this viral cysteine protease, nirmatrelvir prevents viral polyprotein cleavage, which is essential for viral replication, thereby stopping the virus from multiplying in infected cells [156]. According to the structural analysis, Nirmatrelvir’s chemical structure is regarded as a peptidomimetic made up of two primary fragments, known as the “western fragment” and “eastern fragment,” connected by an amide bond. The eastern segment is a γ-lactam analog of Gln with the terminal electrophilic warhead, whereas the western fragment is a bicyclic proline residue linked to an *L*-tert-leucine capped with a trifluoroacetyl group [157]. Ritonavir is an HIV protease inhibitor originally developed for antiretroviral therapy. In the Paxlovid combination, its primary role is pharmacokinetic boosting rather than direct antiviral activity. Ritonavir inhibits CYP3A4, the liver enzyme responsible for metabolizing nirmatrelvir, thereby increasing nirmatrelvir plasma concentrations and prolonging its activity [158,159]. Technically, ritonavir can be described as an L-valinamide derivative where the alpha-amino group is acylated. The synthetic route used for the first kilogram-scale manufacture of nirmatrelvir utilizes HATU/DIPEA and EDC as coupling reagents [160]. The synthesis of ritonavir fundamentally relies on a convergent synthetic strategy. The process constructs a C2-symmetric-like core diamino alcohol backbone, which is subsequently modified with two distinct heterocyclic thiazole arms via carbamate and amide couplings [161].

#### 2.3.9. Fezolinetant

**Figure 38:**
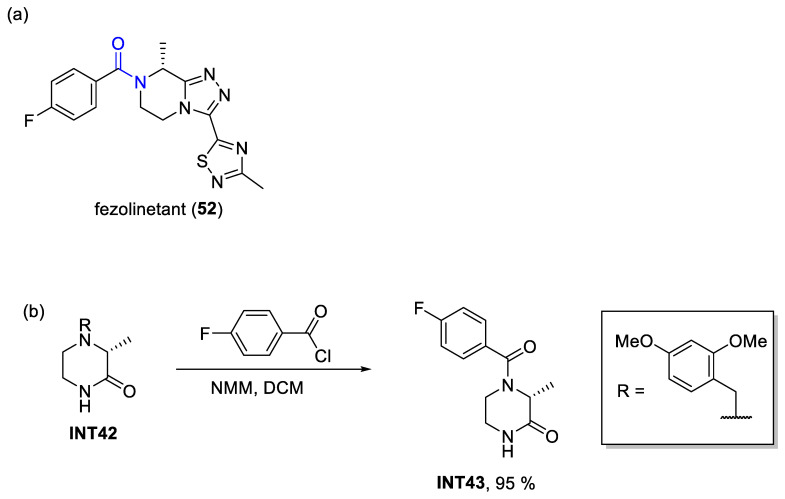
(**a**) Chemical structure of fezolinetant. (**b**) Preparation of **INT43**, used in the synthesis of fezolinetant.

Approved on 12 May 2023, fezolinetant (**52**, brand name Veozah^®^, Figure 38a) is a novel, non-hormonal prescription medication approved to treat moderate-to-severe vasomotor symptoms (hot flashes and night sweats) associated with menopause. It acts as a neurokinin 3 (NK3) receptor antagonist, targeting the brain’s thermoregulatory center to reduce the frequency and severity of hot flashes [162,163]. It contains a triazolopiperazine scaffold endowed with an amide functional group. The tertiary amide is formed via acylation of **INT42** with 4-fluorobenzoyl chloride to give **INT43,** accompanied by simultaneous deprotection of the dimethoxybenzyl group, in excellent yield (95%) [164].

#### 2.3.10. Zavegepant

**Figure 39:**
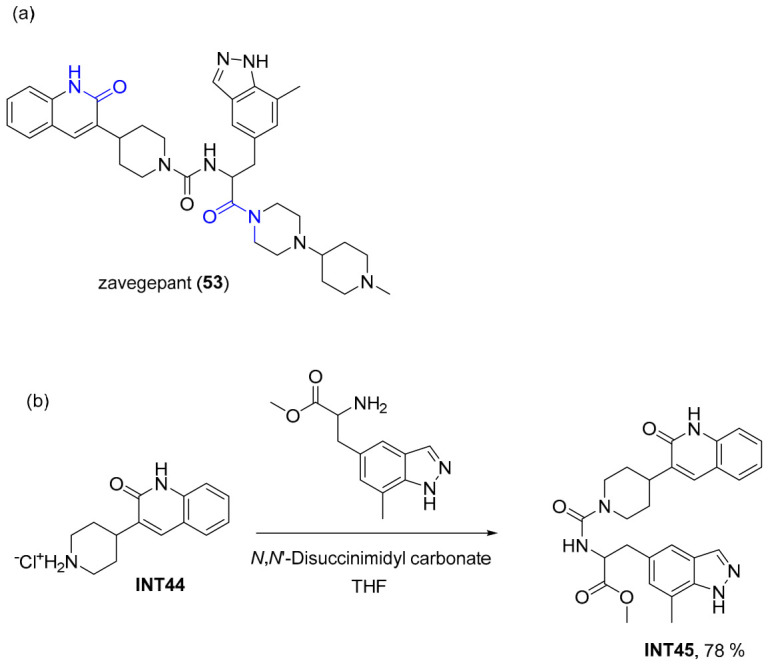
(**a**) Chemical structure of zavegepant. (**b**) Preparation of **INT45**, used in the synthesis of zavegepant.

Approved on 10 March 2023, zavegepant (**53**, brand name Zavzpret^®^, Figure 39a) is the first and only calcitonin gene-related peptide (CGRP) receptor antagonist for the acute treatment of migraine with or without aura in adults [165,166]. Zavegepant does not carry a boxed warning, recommend routine pretreatment or special monitoring, or require a Risk Evaluation and Mitigation Strategy program [166]. It contains a lactam and a classic amide moiety. The synthesis of the urea intermediate for the preparation of zavegepant is presented in Figure 39b. This reaction demonstrates the use of *N*,*N*’-disuccinimidyl carbonate (DSC) as a coupling reagent to form an asymmetric urea bond. The DSC activates either the secondary amine of the piperidine hydrochloride salt (**INT44**) or the primary amine of the phenylalanine-derived indazole ester, allowing them to couple efficiently in THF. This reaction successfully links the two complex building blocks together to yield the urea product **INT45** in a high yield of 78% [127].

#### 2.3.11. Omaveloxolone

**Figure 40:**
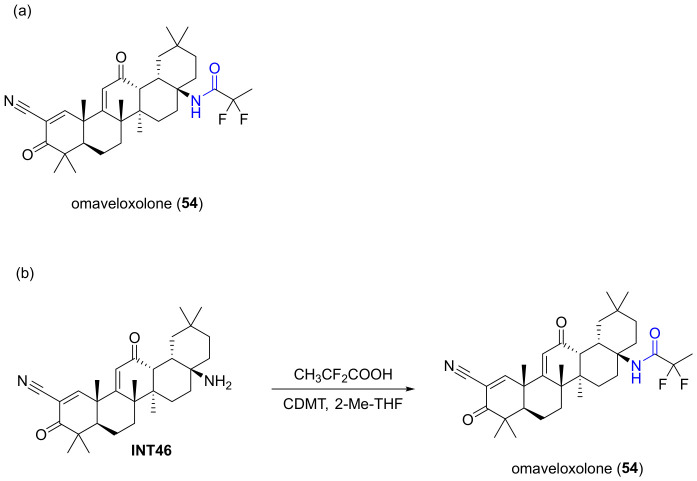
(**a**) Chemical structure of omaveloxolone. (**b**) Final step preparation of omaveloxolone.

Approved on 28 February 2023, omaveloxolone (**54**, brand name Skyclarys^®^, Figure 40a) is a landmark medication, primarily recognized as the first and only drug approved to treat Friedreich’s Ataxia (FA) [167,168]. It serves as a potent activator of the NRF2 pathway, which is critical for restoring mitochondrial function and reducing the oxidative stress that characterizes this rare, progressive genetic disorder [169]. It is an oleanane triterpenoid; it has some structural similarities to steroids with some differences (both C3 and C28 are highly unreactive to substitution reactions) [170]. The synthesis (Figure 40b) utilizes 2-chloro-4,6-dimethoxy-1,3,5-triazine (CDMT) as a coupling reagent to facilitate the amide bond formation between the sterically hindered primary amine of **INT46** and 2,2-difluoropropionic acid. CDMT activates the carboxylic acid in 2-Me-THF to allow efficient acylation at the complex, multi-cyclic core’s amine position. This direct coupling successfully completes the late-stage synthesis of the therapeutic drug omaveloxolone (**54**) [170].

#### 2.3.12. Daprodustat

**Figure 41:**
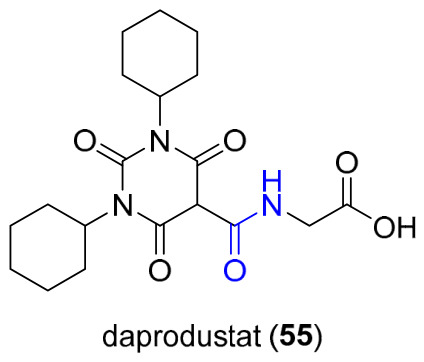
Chemical structure of daprodustat.

Approved by the U.S. Food and Drug Administration (FDA) in February 2023, daprodustat (**55**, brand name Jesduvroq^®^, Figure 41) is a once-daily oral medication for treating anemia caused by chronic kidney disease (CKD) in adults who have been on dialysis for at least four months. It is the first oral Hypoxia-Inducible Factor Prolyl Hydroxylase Inhibitor (HIF-PHI) approved in the U.S. [171]. It is characterized as a barbituric acid core substituted by two cyclohexyl groups and a glycine-derived side chain. Daprodustat’s solubility is governed by the need to balance the solvation of its polar glycine-carboxylic acid arm with its large, hydrophobic dicyclohexyl-barbiturate core. The chemical synthesis of daprodustat focuses on constructing its core barbituric acid derivative structure and coupling it with a glycine moiety [172].

#### 2.3.13. Pirtobrutinib

**Figure 42:**
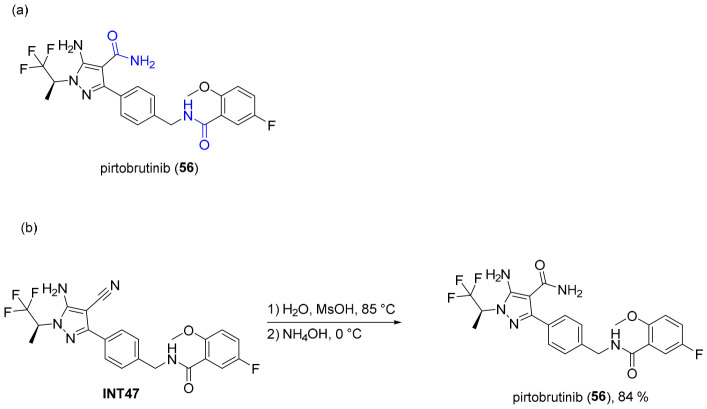
(**a**) Chemical structure of pirtobrutinib. (**b**) Final step preparation of pirtobrutinib.

Approved on 27 January 2023, pirtobrutinib (**56**, brand name Jaypirca^®^, Figure 42a) is a small molecule for the treatment of adults with relapsed or refractory mantle cell lymphoma (MCL) after at least two prior systemic therapies that include a BTK inhibitor [173,174]. This approval was based on response rates observed in clinical trials rather than long-term outcomes. Unlike covalent inhibitors that bind to the Cys481 residue in the ATP-binding pocket, pirtobrutinib interacts with other residues, allowing it to inhibit BTK even when C481 is mutated [175]. Pirtobrutinib is a large, aromatic, heterocycle-rich small molecule with multiple heteroatoms (N, O), several aromatic rings, and limited ionizable functionality under neutral conditions. The last-stage modification (Figure 42b) features the acid-catalyzed partial hydrolysis of the nitrile group on **INT47** using methanesulfonic acid and water. The reaction converts the electron-deficient nitrile directly into a primary amide functionality without disrupting the sensitive pyrazole or benzoxazinone rings. Subsequent neutralization with ammonium hydroxide delivers the targeted BTK inhibitor pirtobrutinib (**56**) in an excellent 84% yield [127].

#### 2.3.14. Trofinetide

**Figure 43:**
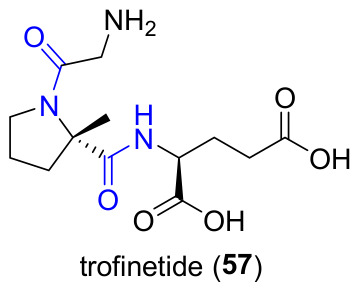
Chemical structure of trofinetide.

Approved on 10 March 2023, trofinetide (**57**, brand name Daybue^®^, Figure 43) is a novel synthetic analog of the naturally occurring tripeptide glycine-proline-glutamate (GPE) designed to address the neurodevelopmental disorder Rett syndrome [176,177]. It modulates neuronal function and is believed to improve synaptic activity and reduce neuroinflammation, although its precise mechanism is not fully understood [178]. Trofinetide is administered orally and is indicated for the treatment of Rett syndrome in both adult and pediatric patients aged 2 years and older [179]. Trofinetide (H-Gly-L-2-MePro-L-Glu-OH) is a synthetic tripeptide analog of glypromate. Its synthesis can be completed using three primary synthetic approaches: Liquid-Phase Peptide Synthesis (LPPS), Solid-Phase Peptide Synthesis (SPPS), and Silylation-Assisted Bulk Synthesis. The primary structural hurdle is coupling the sterically hindered, non-natural amino acid L-2-methylproline (2-MePro) to L-glutamic acid and glycine [180].

#### 2.3.15. Rezafungin

**Figure 44:**
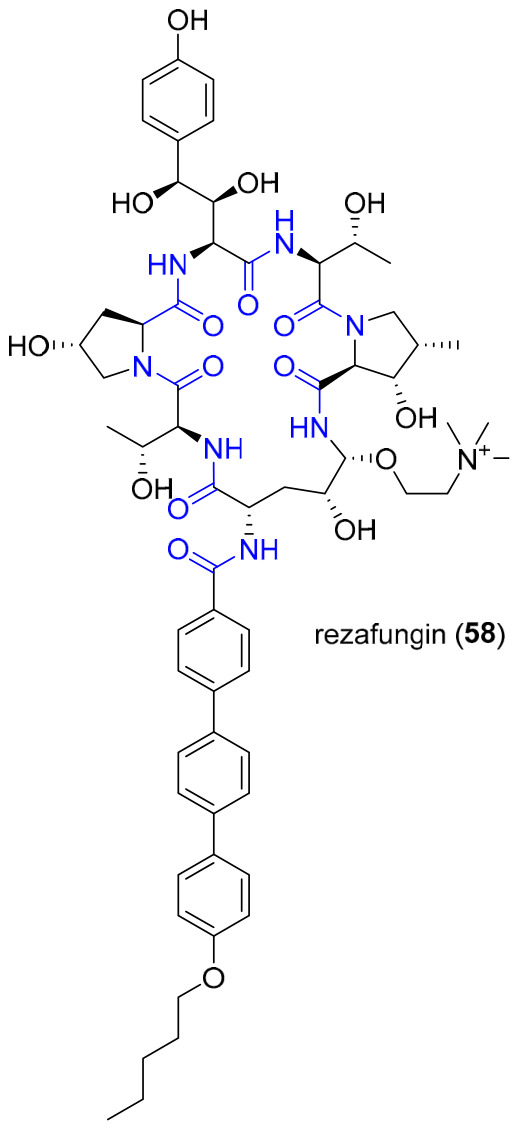
Chemical structure of rezafungin.

Approved on 22 March 2023, rezafungin (**58**, brand name Rezzayo^®^, Figure 44) is a next-generation echinocandin antifungal medication developed for the treatment of candidemia and invasive candidiasis in adults with limited or no alternative treatment options [181]. It works by inhibiting 1,3-β-D-glucan synthase, an enzyme essential for fungal cell wall synthesis, weakening the fungal cell wall and restricting fungal growth and spread [182,183]. Unlike many older echinocandins, rezafungin’s chemical and pharmacokinetic properties support once-weekly intravenous dosing, which can simplify therapy compared to daily regimens [184]. It is a cyclic hexapeptide echinocandin antifungal analog of anidulafungin, characterized by a unique choline aminal ether moiety at the C5 ornithine position. It is a semi-synthetic drug. Its production relies on a hybrid process: the massive, highly complex cyclic peptide core is generated biologically via fungal fermentation, while the structural modifications that give the drug its long-acting stability are added via chemical synthesis [185].

### 2.4. FDA-Approved Drugs in 2024 Containing Amide Group

In 2024, the FDA approved a total of 50 drugs, of which 34 were new chemical entities [28]. As such, 2024 has been an interesting year from the viewpoint of a medicinal chemist, with the approval of two deuterated medications; this class of chemicals now has four approvals overall [28]. Moreover, *N*-aromatic heterocycles and fluorine atoms are present in two-thirds of all the small molecules approved in the year [28].

#### 2.4.1. Alyftrek^®^

**Figure 45:**
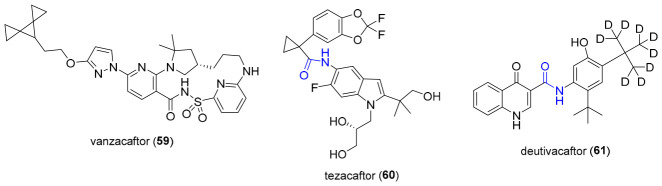
Chemical structure of vanzacaftor, tezacaftor, and deutivacaftor.

The FDA approved Vertex Pharmaceuticals’ Alyftrek^®^ (vanzacaftor **59**/tezacaftor **60**/deutivacaftor **61**, Figure 45) on 20 December 2024, for treating cystic fibrosis in patients aged 6 and older with at least one F508del mutation or other responsive mutations [186]. This once-daily, next-in-class triple-combination modulator offers a new, effective treatment option to enhance lung function. Vanzacaftor is a next-generation cystic fibrosis transmembrane conductance regulator (CFTR) corrector that improves folding and trafficking of the defective CFTR protein to the cell surface in cystic fibrosis. It is designed to act synergistically with other CFTR modulators to enhance functional CFTR expression [187,188,189]. Tezacaftor is a CFTR corrector that increases the amount of properly processed CFTR protein delivered to the cell membrane, particularly in patients with the F508del mutation. It is commonly used in combination regimens to improve chloride transport [190,191]. Deutivacaftor is a deuterated CFTR potentiator that enhances the opening probability of CFTR channels at the cell surface while offering improved pharmacokinetics and a longer duration of action compared with ivacaftor. It helps sustain CFTR activity when used alongside CFTR correctors [190]. The synthesis of tezacaftor concludes with a critical amide bond coupling that joins the cyclopropanecarboxylic acid core to the aminoindole fragment [192,193]. The synthesis of deutivacaftor is accomplished by a convergent amide coupling between an unlabelled quinoline core fragment and a highly deuterated (d_9_) aminophenol core fragment [194].

#### 2.4.2. Ensartinib

**Figure 46:**
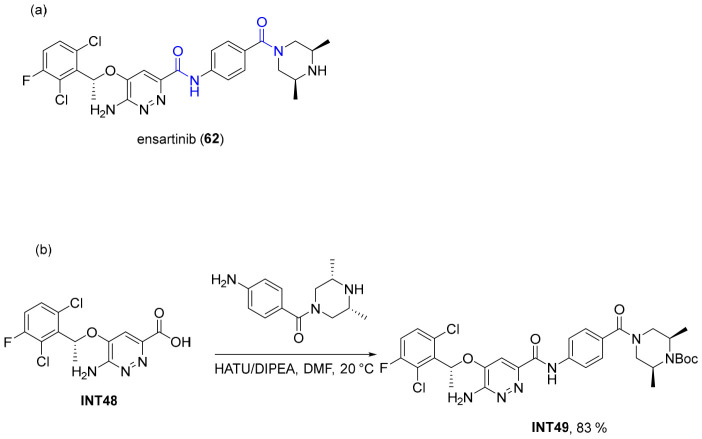
(**a**) Chemical structure of ensartinib. (**b**) Preparation of **INT49**, used in the synthesis of ensartinib.

Approved on 18 December 2024, ensartinib (**62**, brand name Ensacove^®^, Figure 46a) is a next-generation oral tyrosine kinase inhibitor (TKI) approved by the FDA for the treatment of ALK-positive non-small-cell lung cancer [195]. It is specifically indicated for adult patients with anaplastic lymphoma kinase (ALK)-positive locally advanced or metastatic non-small-cell lung cancer (NSCLC) who have not previously received an ALK-inhibitor [196]. ESB’s chemical structure contains a dichloro-fluorophenyl ring and cyclic tertiary amine rings (piperazine). Ensartinib’s solubility is primarily driven by its large, hydrophobic heterocyclic structure. As per the synthesis (Figure 46b), the carboxylic acid of **INT48** is coupled with the primary aniline amine of the coregent using the popular peptide coupling reagents HATU and DIPEA in DMF at 20 °C to yield an amide bond on INT49 in an excellent 83% yield [194].

#### 2.4.3. Iomeprol

**Figure 47:**
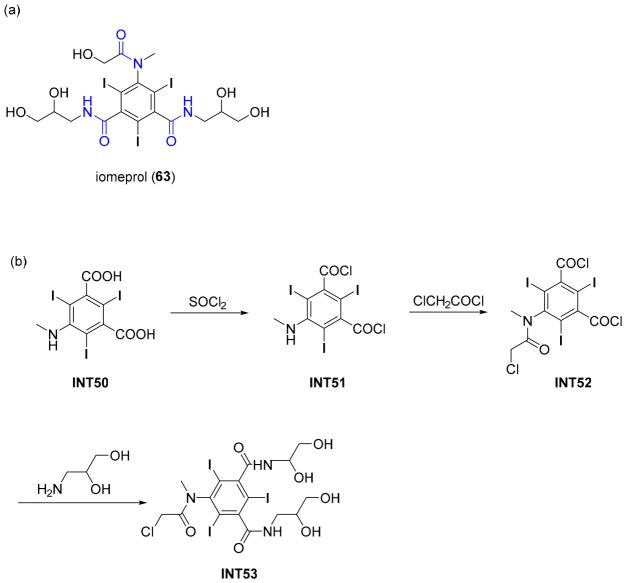
(**a**) Chemical structure of iomeprol. (**b**) Preparation of **INT53**, used in the synthesis of iomeprol.

Approved on 27 November 2024, iomeprol (**63**, brand name Iomervu^®^, Figure 47a) is a nonionic, iodinated radiographic contrast agent for intra-arterial and intravenous procedures in both adult and pediatric patients [197]. Developed by Bracco Diagnostics Inc., it is used to enhance visualization during various diagnostic imaging procedures, including CT scans and arteriography [198]. At the 1 and 3 positions, there are two carbamoyl groups (amide side chains) substituted with 2,3-dihydroxypropyl groups. As per the synthesis (Figure 47b) [199], the dicarboxylic acid **INT50** is treated with thionyl chloride to convert both carboxylic acid groups into highly reactive acyl chlorides, yielding **INT51**. Next, the relatively hindered secondary aniline nitrogen is selectively acylated using chloroacetyl chloride to introduce the chloroacetamide arm in **INT52**. Finally, addition of the aminodiol nucleophile results in chemoselective amidation at the two aromatic acyl chloride positions, leaving the alkyl chloride untouched to yield the highly hydrophilic, iodinated target intermediate **INT53**.

#### 2.4.4. Revumenib

**Figure 48:**
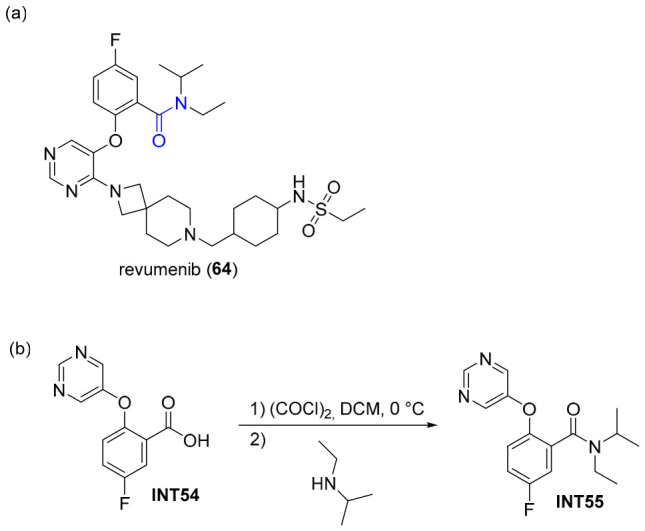
(**a**) Chemical structure of revumenib. (**b**) Preparation of **INT55**, used in the synthesis of revumenib.

Approved on 15 November 2024, revumenib (**64**, brand name Revuforj^®^, Figure 48a) is a first-in-class, oral menin inhibitor approved by the FDA for the treatment of specific, difficult-to-treat types of acute leukemia [200,201]. It works by blocking the interaction between the menin protein and KMT2A, reducing leukemogenic gene expression [202]. It is a complex multi-ring system, including a diazaspiro [3.5]nonane core and a sulfonamide group. The synthesis (Figure 48b) involves the carboxylic acid group of **INT54** being converted into a highly reactive acyl chloride intermediate using oxalyl chloride in dichloromethane (DCM) at 0 °C. In the subsequent step, the secondary amine *N*-ethylisopropylamine is introduced to attack this activated electrophilic intermediate, successfully yielding the tertiary amide **INT55** [203].

#### 2.4.5. Sulopenem Etzadroxil

**Figure 49:**
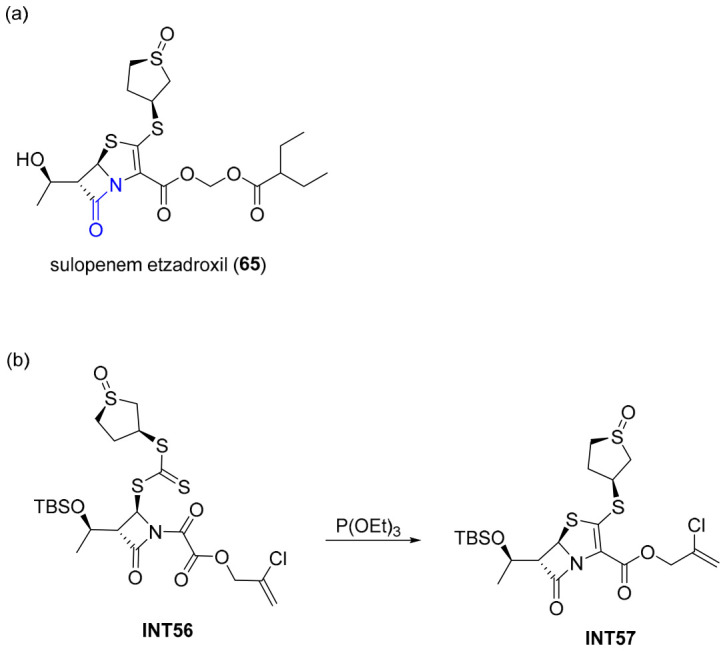
(**a**) Chemical structure of sulopenem etzadroxil. (**b**) Preparation of **INT57**, used in the synthesis of sulopenem etzadroxil.

Sulopenem etzadroxil **65**/probenecid **66**, marketed as Orlynvah^®^ (Figure 49a), is an oral fixed-dose antibiotic combination that the U.S. Food and Drug Administration approved on 25 October 2024 for the treatment of uncomplicated urinary tract infections (uUTIs) caused by *Escherichia coli*, *Klebsiella pneumoniae*, or *Proteus mirabilis* in adult women who have limited or no alternative oral antibacterial treatment options [204,205]. It combines the pro-drug sulopenem etzadroxil (a broad-spectrum penem antibacterial) with probenecid (which increases sulopenem systemic exposure by inhibiting renal tubular secretion) [206]. Sulopenem is a thiopenem, characterized by a sulfur atom in the five-membered ring adjacent to the beta-lactam ring. **65** (the prodrug form of sulopenem) is created by joining an etzadroxil group (ethyl 2-ethylbutanoate) to the carboxylic acid at the C2 position [207]. The synthesis (Figure 49b) proceeds via starting material **INT56,** which contains an unstable trithiocarbonate group attached to the beta-lactam ring. When treated with triethyl phosphite, the trivalent phosphorus nucleophilically attacks the thiocarbonyl sulfur atom. This leads to the extrusion of the central sulfur atom as a phosphite sulfide byproduct, effectively converting the trithiocarbonate group into a transient, highly reactive sulfur-stabilized carbanion or ylide intermediate. This reactive carbon center then immediately attacks the neighboring oxalyl carbonyl group attached to the nitrogen atom, closing the five-membered ring. This tandem sulfur-abstraction and intramolecular cyclization sequence is a classic methodology used in the synthesis of advanced beta-lactam antibiotics, successfully generating the penem core structure found in **INT57** [208].

#### 2.4.6. Inavolisib

**Figure 50:**
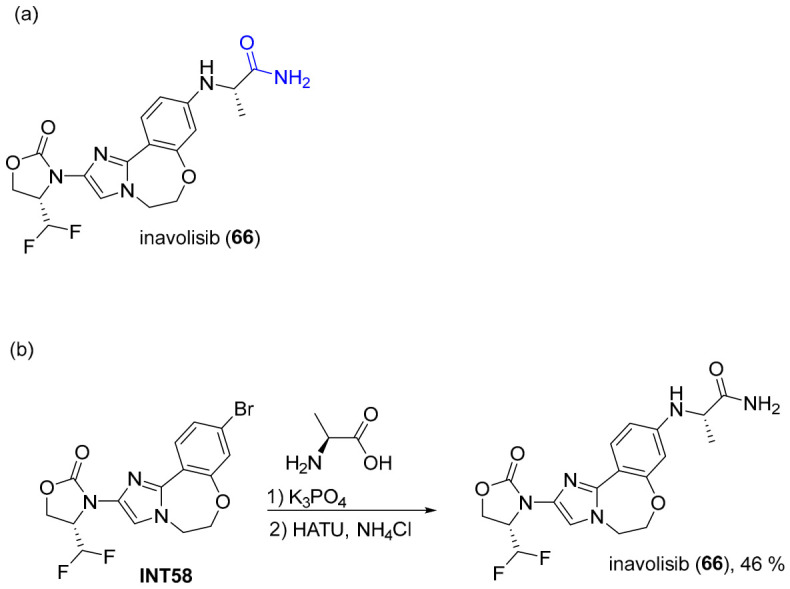
(**a**) Chemical structure of inavolisib. (**b**) Final step preparation of inavolisib.

Approved in October 2024, inavolisib (**66**, brand name Itovebi^®^, Figure 50a) is a first-in-class targeted therapy used to treat specific types of advanced breast cancer. It is primarily prescribed for adults with HR-positive, HER2-negative breast cancer that has a PIK3CA mutation [209]. In inavolisib, a carbamate links part of the heterocyclic system (via an oxygen) to a nitrogen-containing moiety. Structurally, it sits within the oxazolidinone-related region of the molecule. It features a primary amide group on the eastern side of the molecule. Inavolisib is neutral and amphiphilic; the synthesis (Figure 50b) involves [210] the nucleophilic displacement of the aryl bromide on **INT58** with the primary amine of L-alanine. Following this successful amination, the resulting free carboxylic acid tether is immediately engaged in a second-step peptide coupling. The activating agent HATU, alongside an ammonium chloride source, converts the carboxylic acid into a primary amide, completing the installation of the amino acid side chain to deliver inavolisib (66) in a 46% yield over the sequence.

#### 2.4.7. Levacetylleucine

**Figure 51:**
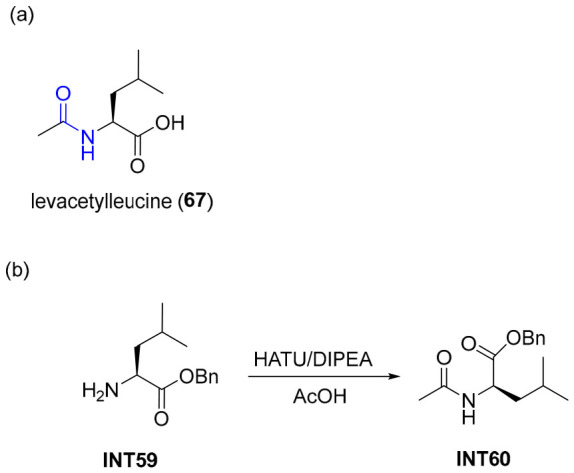
(**a**) Chemical structure of levacetylleucine. (**b**) Preparation of **INT60**, used in the synthesis of levacetylleucine.

Approved on 24 September 2024, levacetylleucine (**67**, brand name Aqneursa^®^, Figure 51a) is also known as *N*-acetyl-*L*-leucine. It is a small-molecule, modified amino acid developed for the treatment of neurological symptoms associated with Niemann–Pick disease type C (NPC), a rare, inherited neurodegenerative disorder [211]. It is the pharmacologically active L-enantiomer of acetylleucine and is administered orally, typically as a suspension [212]. Although its precise mechanism of action is not fully defined, levacetylleucine is thought to improve neuronal function by modulating cellular energy metabolism and stabilizing neuronal signaling, leading to improvements in gait, coordination, and other neurological manifestations seen in NPC [213]. The primary amine of L-leucine benzyl ester (**INT59**) is acetylated by reacting directly with acetic acid. Typically, simple carboxylic acids like acetic acid require heating or harsher conditions to form amides with amines, but the addition of the potent peptide coupling reagent HATU along with the non-nucleophilic base DIPEA efficiently activates the acetic acid in situ. This allows the amine to nucleophilically attack the activated acyl intermediate smoothly at mild temperatures, yielding the protected, acetylated amino acid product **INT60** [194].

#### 2.4.8. Lazertinib

**Figure 52:**
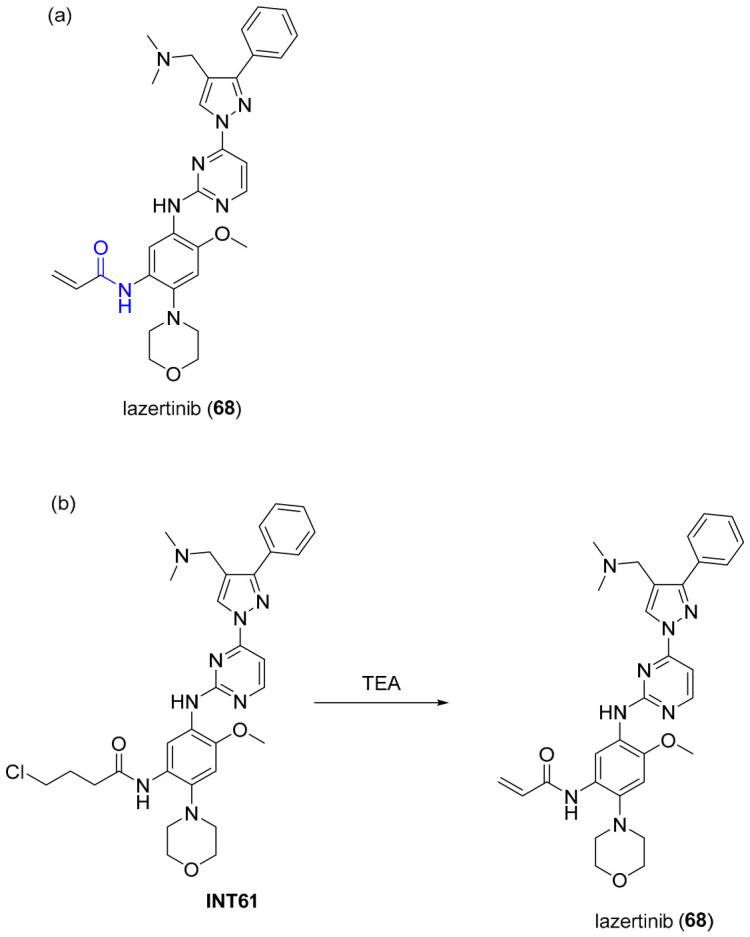
(**a**) Chemical structure of lazertinib. (**b**) Final step preparation of lazertinib.

Approved on 19 August 2024, lazertinib (**68**, brand name Lazcluze^®^, Figure 52a) is a targeted cancer therapy, a third-generation epidermal growth factor receptor (EGFR) tyrosine kinase inhibitor (TKI) [214]. It blocks EGFR signaling in cancer cells, slowing their growth and survival [214]. It is designed to penetrate the brain and target common EGFR mutations seen in non-small-cell lung cancer (NSCLC) [215]. Its structure reflects classic kinase-inhibitor design principles, featuring a heteroaromatic core that fits into the ATP-binding pocket of EGFR, substituents that enhance mutant selectivity and binding affinity. It features a substituted pyrazole core, crucial for selectivity and binding [216], with a phenyl group at R1 and a morpholine-containing side chain for improved pharmacokinetics [216]. It contains an acrylamide functional group that acts as a covalent warhead. As per the synthesis [217], under basic conditions using triethylamine (TEA), the 4-chlorobutanamide side chain of **INT61** undergoes a base-mediated elimination of hydrochloric acid (HCl) to install the terminal alkene, yielding the characteristic acrylamide (Michael acceptor) warhead found in lazertinib.

#### 2.4.9. Tovorafenib

**Figure 53:**
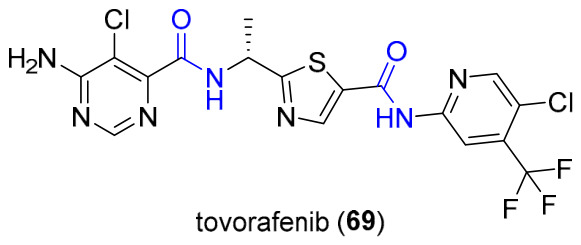
Chemical structure of tovorafenib.

Approved on 23 April 2024, tovorafenib (**69**, brand name Ojemda^®^, Figure 53) is an oral, once-weekly, type II RAF kinase inhibitor developed for the treatment of pediatric brain tumors driven by alterations in the MAPK signaling pathway [218,219]. It targets both mutant and wild-type RAF kinases, including BRAF V600 mutations, as well as BRAF fusions and rearrangements, helping to suppress tumor growth in BRAF-altered cancers [220]. Tovorafenib is indicated for patients aged 6 months and older with relapsed or refractory pediatric low-grade glioma harboring these BRAF alterations [219]. Unlike covalent inhibitors, tovorafenib does not contain a reactive warhead such as an acrylamide; instead, it relies on high-affinity, reversible binding driven by hydrogen bonding, π–π interactions, and hydrophobic contacts. However, the thiazole amide is an important structural feature that contributes to both binding and drug-like properties. The compound has moderate polarity with heteroatoms (for example, amide, thiazole, aromatic systems) but limited hydrogen-bond donation. The amide bonds in tovorafenib are generated via oxalyl chloride and the EDC/HOBt coupling reagent [221].

#### 2.4.10. Ceftobiprole Medocaril Sodium

**Figure 54:**
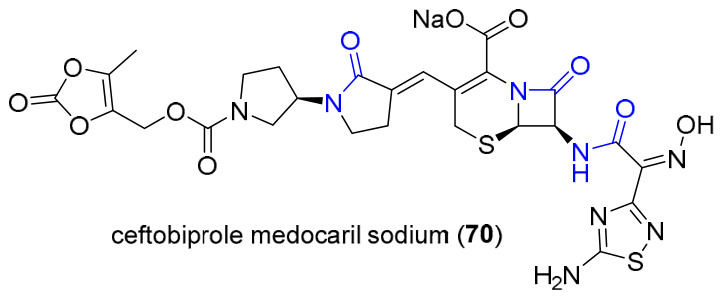
Chemical structure of ceftobiprole medocaril sodium.

Approved on 3 April 2024, ceftobiprole medocaril sodium (**70**, brand name Zevtera^®^, Figure 54) is a fifth-generation cephalosporin antibiotic developed as the prodrug of ceftobiprole, designed for intravenous use and active against a broad spectrum of Gram-positive and some Gram-negative bacteria, including *methicillin-resistant Staphylococcus aureus* (MRSA) [222]. It works by binding to multiple penicillin-binding proteins (PBPs), inhibiting bacterial cell wall synthesis, and exerting rapid bactericidal activity against susceptible pathogens [223,224]. The medocaril moiety improves water solubility and prodrug delivery, with conversion in vivo to the active antibiotic ceftobiprole [225]. Its core structure is the characteristic cephalosporin nucleus, which consists of a four-membered β-lactam ring fused to a six-membered dihydrothiazine ring. This bicyclic scaffold is essential for antibacterial activity, as it enables binding to bacterial penicillin-binding proteins (PBPs) and inhibition of cell wall synthesis once the active drug is released.

#### 2.4.11. Danicopan

**Figure 55:**
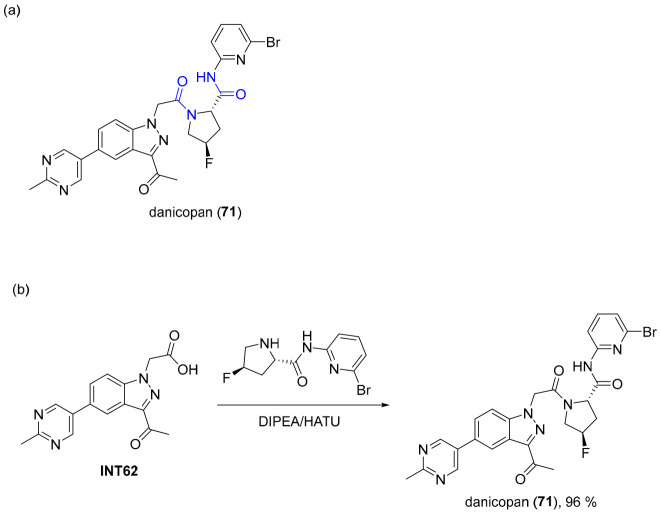
(**a**) Chemical structure of danicopan. (**b**) Final step preparation of danicopan.

Approved on 29 March 2024, danicopan (**71**, brand name Voydeya^®^, Figure 55a) is an add-on treatment to C5 inhibitors for adults with paroxysmal nocturnal hemoglobinuria (PNH) who have persistent extravascular hemolysis. It is an oral complement factor D inhibitor designed to improve hemoglobin levels in patients inadequately controlled on standard C5 inhibitor therapy [226]. The (2-methylpyrimidin-5-yl) group at the 5-position of the indazolyl moiety gives a biheteroaryl framework. This group imparts improved potency to danicopan when compared with the unsubstituted analog [227]. Synthetically, the carboxylic acid of the indazole intermediate **INT62** is coupled with the secondary amine of the fluoropyrrolidine core using the peptide coupling reagent HATU and the non-nucleophilic base DIPEA, constructing the central amide bond with an excellent 96% yield (Figure 55b) [227].

#### 2.4.12. Vadadustat

**Figure 56:**
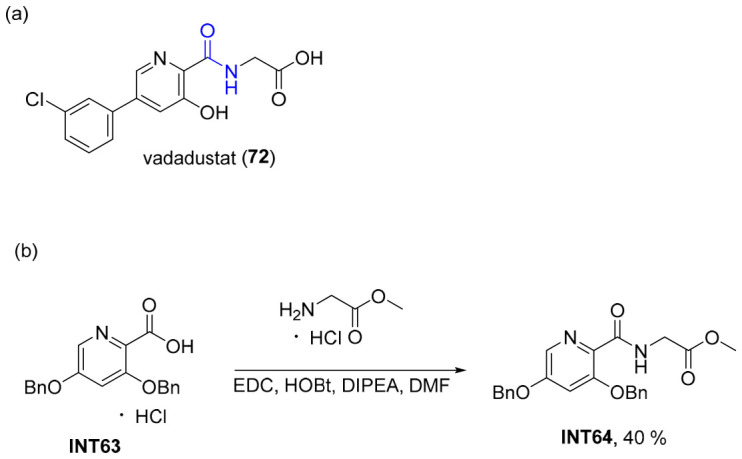
(**a**) Chemical structure of vadadustat. (**b**) Preparation of **INT64**, used in the synthesis of vadadustat.

Approved in March 2024, vadadustat (**72**, brand name Vafseo^®^, Figure 56a) is an oral hypoxia-inducible factor prolyl hydroxylase (HIF-PH) inhibitor used to treat anemia due to chronic kidney disease (CKD) in adults who have been on dialysis for at least three months [228]. It works by stabilizing HIF to increase the body’s production of erythropoietin and promote red blood cell formation. Vadadustat binds the catalytic site of PHD2, resulting in bidentate chelation interactions with the active site Fe atom, a salt bridge interaction with Arg383 deep in the binding pocket, an H-bond interaction of the vadadustat phenolic OH with Tyr303, and nonspecific lipophilic interactions of the chlorophenyl ring with residues near the HIF binding site [229]. The carboxylic acid of the pyridine intermediate **INT63** is coupled with the amine of the glycine core using the peptide coupling reagent EDC and the non-nucleophilic base DIPEA, constructing the central amide bond of **INT64** with an excellent 40% yield (Figure 56b) [230].

#### 2.4.13. Resmetirom

**Figure 57:**
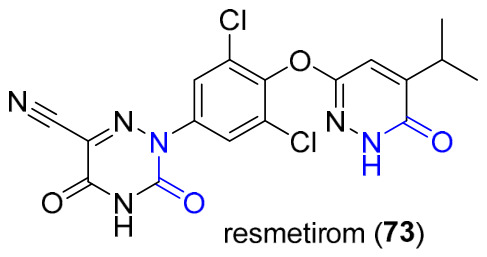
Chemical structure of resmetirom.

Approved in March 2024, resmetirom (**73**, brand name Rezdiffra^®^, Figure 57) is an oral, liver-directed thyroid hormone receptor-β agonist for adults with noncirrhotic nonalcoholic steatohepatitis (NASH), also known as metabolic dysfunction-associated steatohepatitis (MASH), with moderate to advanced fibrosis (F2–F3), to be used alongside diet and exercise [231,232]. It is the first FDA-approved medication specifically indicated for NASH/MASH with fibrosis. Resmetirom works by selectively activating THR-β in the liver, reducing hepatic fat accumulation, inflammation, and fibrosis progression [233]. Chemically, it is a non-steroidal, triiodothyronine (T3)-mimetic derivative, designed to retain the lipid-lowering and metabolic effects of thyroid hormone in the liver while reducing systemic exposure [234]. It can be synthesized via an optimized, highly efficient four-step streamlined process. This route replaces older, lengthier patent processes by utilizing a clever “one-pot” tandem cyclization and hydrolysis sequence, achieving an overall yield of 44.3% and a purity of 98.8% [235].

#### 2.4.14. Cefepime/Enmetazobactam

**Figure 58:**
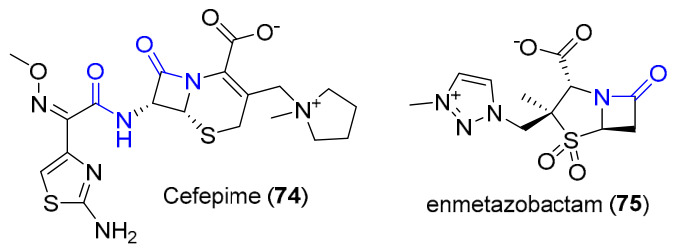
Chemical structure of cefepime/enmetazobactam.

Cefepime **74**/enmetazobactam **75**, marketed under the brand name Exblifep^®^ (Figure 58), was approved by the FDA in February 2024. The product was developed by Allecra Therapeutics and is indicated for the treatment of complicated urinary tract infections, including pyelonephritis, in adults and certain pediatric patients caused by susceptible Gram-negative microorganisms [236]. Cefepime is a fourth-generation cephalosporin antibiotic that inhibits bacterial cell wall synthesis. Enmetazobactam is a novel β-lactamase inhibitor that protects cefepime from degradation by certain β-lactamases, including extended-spectrum β-lactamases (ESBLs) [237]. The combination restores and enhances activity against resistant Enterobacterales, offering an important therapeutic option in settings where antimicrobial resistance is a concern [238]. The FDA approval was supported by results from a Phase 3, randomized, double-blind clinical trial comparing cefepime/enmetazobactam with piperacillin/tazobactam in patients with complicated urinary tract infections. The study demonstrated statistical superiority in overall treatment success and higher microbiological eradication rates, with a safety profile generally consistent with other β-lactam antibiotics [239]. The drug is administered intravenously, and dose adjustments are required in patients with renal impairment [240]. Common adverse reactions include infusion site reactions, gastrointestinal symptoms such as diarrhea and nausea, headache, and potential hypersensitivity reactions typical of β-lactam antibiotics [241]. Overall, cefepime/enmetazobactam represents a significant addition to the antimicrobial armamentarium, particularly for infections caused by ESBL-producing Gram-negative bacteria, and may help reduce reliance on carbapenem therapy in appropriate patients [242]. Cefepime is derived from the cephalosporin nucleus, 7-aminocephalosporanic acid. Chemically, it contains the characteristic β-lactam ring fused to a dihydrothiazine ring that defines cephalosporins. Enmetazobactam is a β-lactamase inhibitor structurally related to tazobactam but modified to improve potency and spectrum. While both are penicillanic acid sulfones (PAS), the structural difference lies in the addition of a methyl group to the triazole moiety of enmetazobactam. For the synthesis of cefepime, 7-amino-3-(l-methylpyrrolidinio)methyl-3-cephem-4-carboxylate is used, which reacts with the activated ester [243].

### 2.5. FDA-Approved Drugs in 2025 Containing Amide Group

In 2025, the FDA approved a total of 46 drugs, of which 34 were new chemical entities [244]. Kinase inhibitors are making steady inroads into inflammatory and immune-mediated diseases, with therapies targeting Bruton’s tyrosine kinase (BTK) at the forefront. Initially approved to treat blood cancers by eliminating malignant B cells, BTK inhibitors are now being leveraged for their ability to fine-tune B-cell activity in autoimmune conditions. This year marked key milestones for the class. Sanofi secured the first approval for its BTK inhibitor rilzabrutinib (**76**), marketed as Wayrilz^®^, to treat immune thrombocytopenia, a rare autoimmune blood disorder [245]. Meanwhile, Novartis won regulatory clearance for remibrutinib (**77**), sold as Rhapsido^®^, for chronic spontaneous urticaria, commonly known as chronic hives [246]. Both drugs contain an amide linker in their skeleton.

#### 2.5.1. Rilzabrutinib

**Figure 59:**
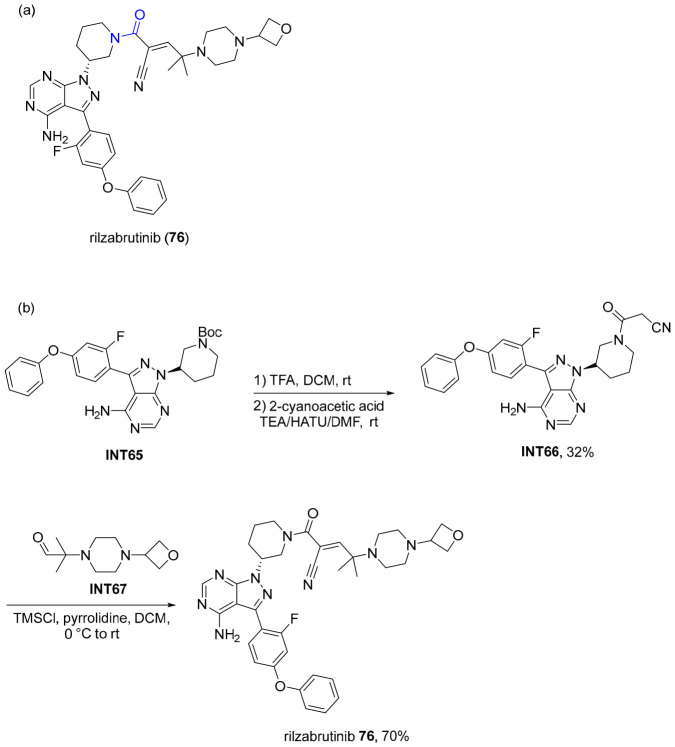
(**a**) Chemical structure of rilzabrutinib. (**b**) Synthetic steps for rilzabrutinib.

Approved on 29 August 2025, rilzabrutinib (**76**, brand name Wayrilz^®^, Figure 59a) is an oral, reversible Bruton’s tyrosine kinase (BTK) inhibitor developed by Sanofi for the treatment of immune-mediated diseases [247]. It works by modulating B-cell and other immune signaling pathways involved in autoimmune disorders [248]. Before approval, rilzabrutinib was granted Fast Track designation in 2020 for immune thrombocytopenia (ITP), followed in 2025 by orphan drug designations for warm autoimmune hemolytic anemia (wAIHA), IgG4-related disease, and sickle cell disease [249]. In February 2026, it also received Breakthrough Therapy designation for wAIHA, reflecting evidence of potential substantial improvement over existing treatments. Rilzabrutinib continues to be studied in additional rare and immune-mediated conditions [250]. Chemically, it is designed as a targeted kinase inhibitor with a structure optimized to bind selectively to the ATP-binding site of BTK. Unlike earlier covalent BTK inhibitors, rilzabrutinib forms a reversible covalent bond with a cysteine residue (Cys481) in the BTK active site, allowing strong target engagement with controlled off-target activity [251,252]. It displays a cyanoacrylamide warhead (Figure 59a). Synthetically, the free piperidine group from INT65 was condensed with cyanoacetic acid using hexafluorophosphate azabenzotriazole tetramethyl uronium (HATU) and TEA in DMF to give amide INT66 (two-step yield: 32%). Eventually, the crucial step to form α-cyanoacrylamide was a Knoevenagel condensation reaction with the aldehyde **INT67** catalyzed by trimethylsilyl chloride (TMSCl) to give the final product rilzabrutinib **76** in good yield (70%, Figure 59b) [253].

#### 2.5.2. Remibrutinib

**Figure 60:**
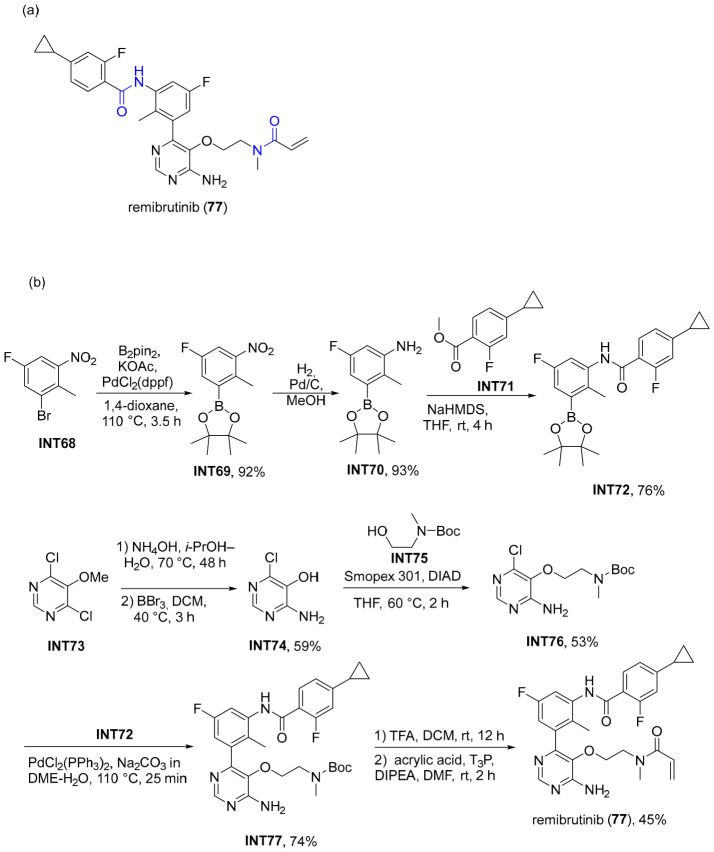
(**a**) Chemical structure of remibrutinib. (**b**) Synthetic steps for remibrutinib.

Approved on 30 September 2025, Remibrutinib (**77**, brand name Rhapsido^®^, Figure 60a) is a small molecule for the treatment of chronic spontaneous urticaria (CSU) in adult patients who remain symptomatic despite H1 antihistamine therapy, marking it as the first BTK inhibitor approved for this allergic skin condition [246]. Remibrutinib is an oral, highly selective, covalent Bruton’s tyrosine kinase (BTK) inhibitor developed by Novartis that targets BTK signaling to reduce immune-mediated release of histamine and other pro-inflammatory mediators in conditions driven by mast cells and basophils [254]. Chemically, it is a small-molecule heterocycle designed for potency, selectivity, and oral bioavailability with a covalent binding mechanism to Cys481 in the BTK active site, giving sustained inhibition with a favorable safety profile compared with less selective agents [255]. The approval was supported by positive Phase III data demonstrating significant improvements in itch and hive severity and overall disease activity compared with placebo in patients with CSU [254]. Remibrutinib is also under investigation in other immune-mediated diseases such as chronic inducible urticaria, hidradenitis suppurativa, and food allergy [256]. It contains an acrylamide moiety. The first synthon is obtained from 1-bromo-5-fluoro-2-methyl-3-nitrobenzene (**INT68**), which underwent a Suzuki coupling using bis (pinacolato)diboron (B_2_pin_2_), potassium acetate (KOAc), and PdCl_2_(dppf) in 1,4-dioxane in excellent yield (**INT69**, 92%). The following reduction (catalytic hydrogenation) of the nitro group afforded aniline **INT70** (93% yield), which was used to prepare amide **INT72** by reacting with methyl ester **INT71** using sodium hexamethyldisilazide (NaHMDS) as the base in THF (76%). The second synthon is obtained via reacting pyrimidine **INT73** with ammonium hydroxide (NH_4_OH) and BBr_3_ to give monochloroaniline **INT74** (59%). This was transformed into tertiary amine **INT75** via a polymer-bound triphenylphosphine (Smopex 310) in the corresponding Mitsunobu reaction involving tert-butyl (2-hydroxyethyl)(methyl)carbamate **INT76** (53%). This reacted with **INT72** under Suzuki conditions [(PdCl_2_(PPh_3_)_2_ Na_2_CO_3_ in 1,2-Dimethoxyethane/water, DME–H_2_O, 110 °C, 25 min)] to give the precursor **INT77** for remibrutinib (**77**). The last step involved the deprotection of the BOC group (using TFA) and the formation of acrylamide via propanephosphonic acid anhydride (T_3_P) in a moderate yield (45%, Figure 60b) [253].

#### 2.5.3. Aficamten

**Figure 61:**
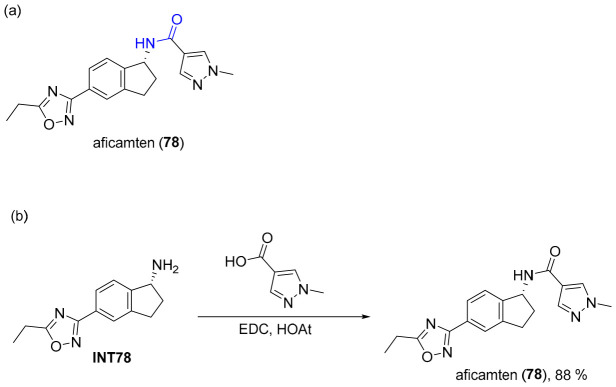
(**a**) Chemical structure of aficamten. (**b**) Synthetic steps for aficamten.

Approved on 19 December 2025, aficamten (**78**, brand name Myqorzo^®^, Figure 61a) is a selective small-molecule inhibitor of cardiac myosin developed to treat symptomatic obstructive hypertrophic cardiomyopathy (HCM), a genetic heart condition in which abnormally thickened heart muscle impedes blood flow and causes symptoms like shortness of breath, chest pain, and fatigue [244,257]. It works by binding to cardiac myosin and reducing excessive cross-bridge formation between actin and myosin during the contractile cycle, thereby lowering hypercontractility and improving left ventricular outflow tract obstruction without abolishing normal systolic function [258]. The synthesis proceeds via the primary amine of the chiral aminoindane intermediate **INT78**, which is coupled with 1-methyl-1*H*-pyrazole-4-carboxylic acid. The transformation utilizes the carbodiimide coupling agent EDC in combination with 1-Hydroxy-7-azabenzotriazole (HOAt) to drive the condensation efficiently, affording the target drug molecule in an 88% yield while preserving the stereochemical integrity of the chiral center (Figure 61b) [259].

#### 2.5.4. Sevabertinib

**Figure 62:**
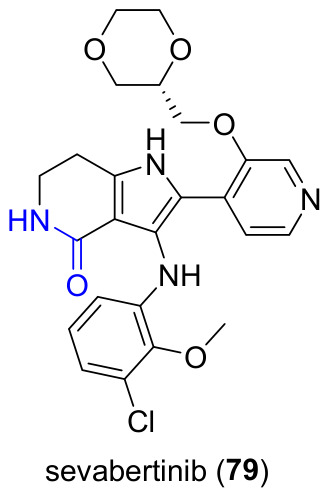
Chemical structure of sevabertinib.

Approved on 19 November 2025, sevabertinib (**79**, brand name **Hyrnuo**^®^, Figure 62) is an investigational, orally available small-molecule pan-ERBB tyrosine kinase inhibitor [260]. It targets members of the ERBB receptor family (including EGFR/HER1, HER2, and HER4) and is being developed primarily for non-small-cell lung cancer (NSCLC) with EGFR exon 20 insertion mutations, which are typically resistant to earlier EGFR inhibitors [261,262]. Chemically, sevabertinib is a multi-ring heteroaromatic small molecule, an ATP-mimetic in design, optimized for kinase hinge binding with balanced lipophilicity and oral delivery. Sevabertinib, like most kinase inhibitors, contains multiple aromatic rings with heterocycles and limited polar surface area, giving it moderate to high lipophilicity with some hydrogen-bonding capacity. The piperidin-2-one-containing building block was introduced intact rather than being generated de novo during the synthesis [253].

#### 2.5.5. Elinzanetant

**Figure 63:**
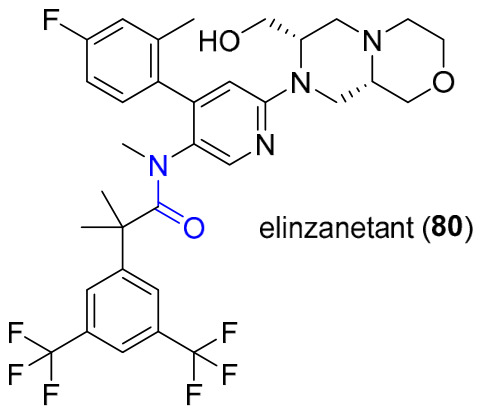
Chemical structure of elinzanetant.

On 24 October 2025, the FDA approved elinzanetant (**80**, brand name Lynkuet^®^, Figure 63) for the treatment of women with moderate-to-severe vasomotor symptoms due to menopause (IPF) [263]. Elinzanetant was discovered through Bayer’s internal neurokinin receptor antagonist program, originally targeting central nervous system pathways involved in thermoregulation and hormone feedback. Optimization efforts focused on developing a compound that could simultaneously block neurokinin-1 (NK1) and neurokinin-3 (NK3) receptors [264,265]—both implicated in menopausal vasomotor symptoms (VMS) such as hot flashes while maintaining favorable pharmacokinetics and safety [266]. The compound was selected for development after promising preclinical results showing potent inhibition of substance P [267] and neurokinin B signaling pathways, with good oral bioavailability and central nervous penetration [268]. It is a drug-like organic compound with multiple aromatic and heteroaromatic rings, nitrogen-containing functional groups, and amide linkages that enable hydrogen bonding and receptor binding. It has a moderate molecular weight and significant lipophilicity, typical of CNS-active agents, allowing membrane permeability while maintaining receptor selectivity. Elinzanetant contains extensive aromatic and aliphatic regions with limited strongly polar or ionizable functionality, so its overall character is largely lipophilic. The patent EP4431512A1 [269] mentions “methods of preparing said compounds, intermediate compounds useful for preparing said compounds” but is somewhat general and is directed to a family of compounds (not exclusively elinzanetant). Therefore, the specific step-by-step synthesis for the marketed molecule may be partly confidential.

#### 2.5.6. Brensocatib

**Figure 64:**
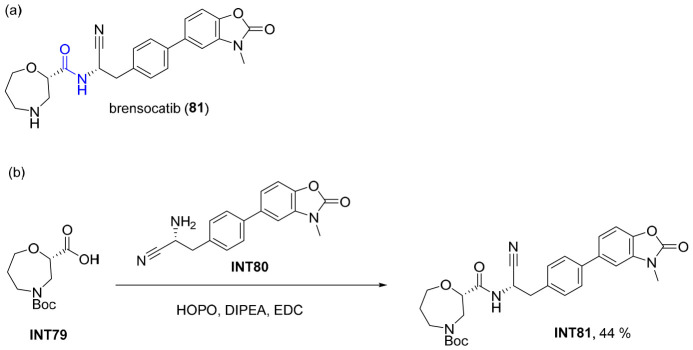
(**a**) Chemical structure of brensocatib. (**b**) Preparation of **INT81**, used in the synthesis of brensocatib.

Approved by the FDA in August 2025, brensocatib (**81**, brand name **Brinsupri**^®^, Figure 64a) is an oral, selective inhibitor of dipeptidyl peptidase 1 (also known as cathepsin C) developed to reduce excessive neutrophil-driven inflammation [270,271]. By blocking activation of neutrophil serine proteases, it aims to limit tissue damage associated with chronic inflammatory lung diseases, particularly non-cystic fibrosis bronchiectasis [272]. Brensocatib was developed by Insmed Incorporated and demonstrated clinically meaningful reductions in pulmonary exacerbations in Phase 3 trials in patients with bronchiectasis [273]. Unlike peptide-based protease inhibitors, brensocatib is structurally stable against rapid enzymatic degradation because it lacks a classical peptide backbone, displaying a 1,4-oxazepane-2-carboxamide backbone. The synthesis (Figure 64b) involves an amide coupling reaction where EDC activates the carboxylic acid (**INT79**) to form a reactive intermediate, which is then converted into a more stable active ester by 2-hydroxypyridine-*N*-oxide (HOPO) to prevent racemization. DIPEA acts as a non-nucleophilic base to free the amine (**INT80**) and neutralize acidic byproducts, facilitating its attack on the active ester to form **INT81** [274].

#### 2.5.7. Zongertinib

**Figure 65:**
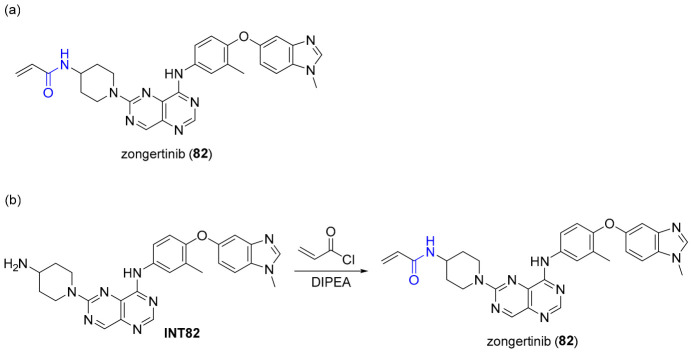
(**a**) Chemical structure of zongertinib. (**b**) Final step preparation of zongertinib.

Approved on 8 August 2025, zongertinib (**82**, brand name Hernexeos^®^, Figure 65a) is an oral, selective HER2 (ERBB2) tyrosine kinase inhibitor developed by Boehringer Ingelheim for the treatment of HER2-mutant non-small-cell lung cancer (NSCLC) [275]. It works by irreversibly inhibiting HER2 signaling, thereby blocking tumor cell growth driven by activating HER2 mutations [276]. The acrylamide moiety in zongertinib acts as a covalent warhead that enables irreversible inhibition of the HER2 kinase domain [277]. In the kinase active site, the electrophilic double bond of the acrylamide reacts with a nucleophilic cysteine residue in HER2 through Michael addition, forming a covalent thioether bond between the inhibitor and the protein [277]. Its synthesis (Figure 65b) proceeds via a primary amine on the piperidine ring of **INT82** that acts as the nucleophile, attacking the electrophilic carbonyl carbon of the acryloyl chloride. Elimination of the chloride leaving group forms the amide bond, while DIPEA acts as an acid scavenger to neutralize the hydrochloric acid generated during the process [278].

#### 2.5.8. Dordaviprone

**Figure 66:**
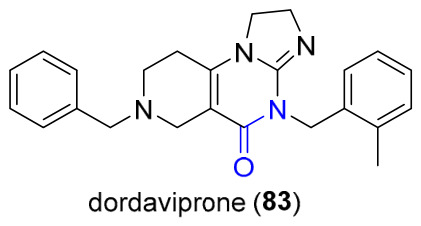
Chemical structure of dordaviprone.

Approved on 6 August 2025, dordaviprone (**83**, brand name Modeyso^®^, Figure 66) is an oral small-molecule imipridone anticancer agent developed by Chimerix that targets mitochondrial protease ClpP and antagonizes the dopamine receptor DRD2, leading to activation of the integrated stress response and induction of tumor cell apoptosis [279]. It is characterized by a tri-heterocyclic imidazopyridone core structure. Imipridones were originally discovered through screening for compounds that selectively induce tumor cell death without strongly affecting normal cells. Its synthesis is achieved via a copper-catalyzed tandem approach. This strategy selectively activates the specific nucleophilic nitrogen site determined by the geometric constraints of the copper–ligand–substrate complex. This explicitly forces an angular cyclization pathway, delivering the thermodynamically favored angular isomer with absolute regiocontrol [280].

#### 2.5.9. Delgocitinib

**Figure 67:**
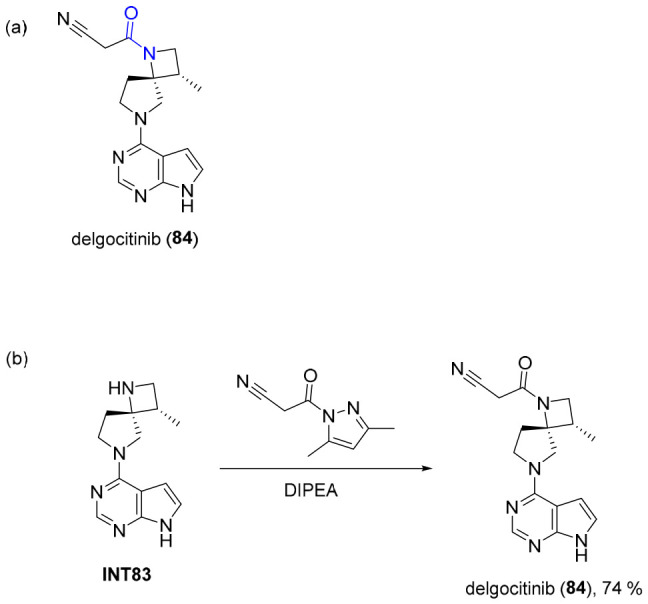
(**a**) Chemical structure of delgocitinib. (**b**) Final step preparation of delgocitinib.

The FDA approved delgocitinib (**84**, brand name Anzupgo^®^, Figure 67a) on 23 July 2025, for treating adults with moderate-to-severe chronic hand eczema [281]. This topical pan-Janus kinase inhibitor serves as a non-steroidal alternative for patients who fail to find relief with traditional corticosteroids. It is designed for twice-daily application and works by blocking the signaling pathways responsible for skin inflammation and itching [282]. The synthesis proceeds via acylation at the azetidine nitrogen (Figure 67b). The transformation is a nucleophilic acyl substitution reaction where the secondary amine of the azetidine ring in **INT83** attacks the carbonyl carbon of the cyanoacetylating reagent, 1-(2-cyanoacetyl)-3,5-dimethyl-1*H*-pyrazole. Non-nucleophilic base DIPEA facilitates the reaction by neutralizing the leaving 3,5-dimethylpyrazole group. This step introduces the specific cyanoacetyl warhead onto the azetidine nitrogen, yielding delgocitinib in 74% yield without perturbing the stereochemistry of the core or the reactive pyrrolopyrimidine scaffold [283].

#### 2.5.10. Sebetralstat

**Figure 68:**
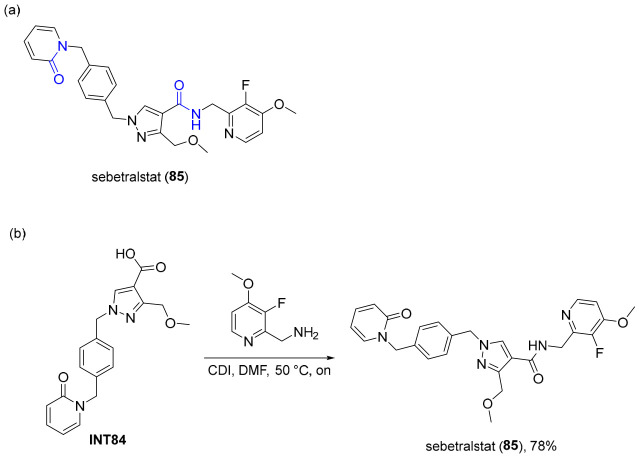
(**a**) Chemical structure of sebetralstat. (**b**) Final step preparation of sebetralstat.

Approved on 7 July 2025 by the FDA [284], sebetralstat (**85**, Ekterly^®^, Figure 68a) is an oral drug developed by KalVista Pharmaceuticals for the on-demand treatment of hereditary angioedema (HAE) attacks [284,285]. Hereditary angioedema (HAE) attacks are characterized by sudden, painful swelling episodes in various parts of the body. These attacks can affect the face, limbs, abdomen, and, most seriously, the airways, potentially leading to life-threatening situations [286]. Sebetralstat is a selective plasma kallikrein inhibitor designed to prevent the excessive production of bradykinin [287]—a peptide that increases vascular permeability and causes the painful, potentially life-threatening swelling characteristic of HAE. The most common side effect of sebetralstat is headache [288]. It features a unique 3-fluoro-4-methoxypyridyl moiety. The transformation is a CDI-mediated amide coupling reaction. The carboxylic acid group on the pyrazole ring of **INT84** is first activated in situ by 1,1′-carbonyldiimidazole (CDI) in DMF at 50 °C to form a highly reactive acyl imidazole intermediate. Subsequent nucleophilic attack by the primary amine of (3-fluoro-4-methoxypyridin-2-yl)methanamine onto this activated carbonyl yields the target compound, sebetralstat, with a 78% chemical yield over the course of an overnight reaction (Figure 68b) [253].

#### 2.5.11. Sunvozertinib

**Figure 69:**
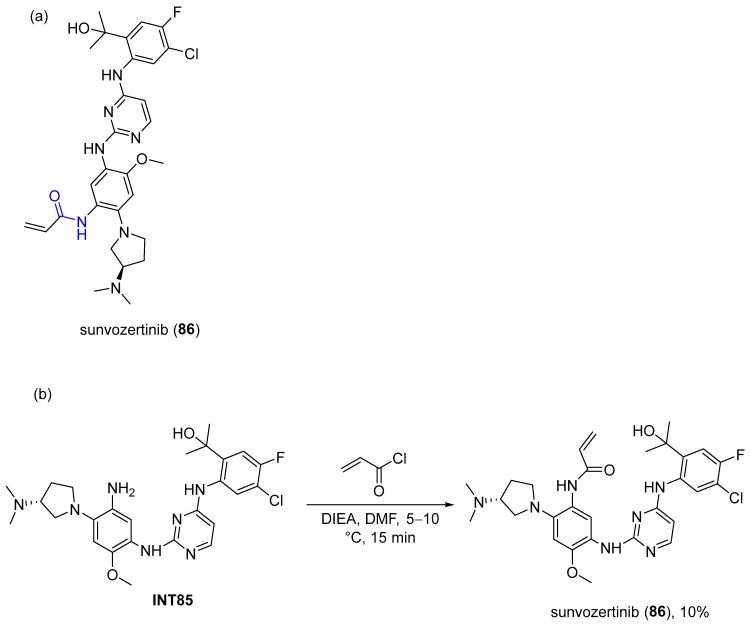
(**a**) Chemical structure of sunvozertinib. (**b**) Final step preparation of sunvozertinib.

Sunvozertinib (**86**, brand name Zegfrovy^®^, Figure 69a) is an oral, irreversible tyrosine kinase inhibitor (TKI) that minimizes action against wild-type EGFR while specifically targeting EGFR exon 20 insertion mutations and specific HER2 exon 20 insertion mutations. In non-small-cell lung cancer (NSCLC) models, it disrupts angiogenesis and tumor cell proliferation by binding covalently to the kinase [289].

On 2 July 2025, the FDA granted expedited clearance for adult patients with advanced or metastatic non-small-cell lung cancer (NSCLC) who have EGFR exon 20 insertions following platinum chemotherapy, as identified by an FDA-approved test [290]. It has one fluorine and one chlorine on the same aromatic ring. The conversion proceeds via a highly regioselective nucleophilic acyl substitution reaction. The primary aniline nitrogen situated on the central benzene ring of **INT85** acts as the nucleophile, targeting the electrophilic carbonyl carbon of acryloyl chloride. The reaction runs efficiently at low temperatures between 5 and 10 °C in DMF for 15 min, driven by DIEA, which acts as a base to scavenge the liberated hydrochloric acid. This amidation process specifically installs the crucial acrylamide group onto the scaffold, providing sunvozertinib with a 10% chemical yield (Figure 69b) [253].

#### 2.5.12. Acoltremon

**Figure 70:**
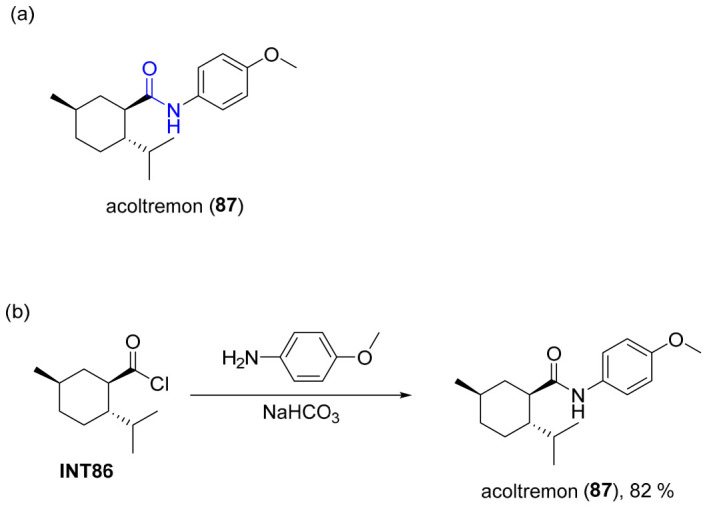
(**a**) Chemical structure of acoltremon. (**b**) Final step preparation of acoltremon.

Acoltremon (**87**, brand name Tryptyr^®^, Figure 70a) was FDA-approved in May 2025 as a first-in-class ophthalmic drug for dry eye disease, acting as a TRPM8 thermoreceptor agonist that stimulates corneal sensory nerves and trigeminal signaling to increase basal tear production with rapid onset, often within one day [291]. Pharmacologically, it is administered topically as a 0.003% ophthalmic solution (one drop per eye twice daily), producing localized effects with minimal systemic exposure, showing good tolerability with mainly instillation-site pain as the common adverse effect, and demonstrating efficacy in Phase III trials through significant increases in tear production [292]. Its synthesis starts from chloride (**INT86**). The transformation is a nucleophilic acyl substitution reaction between a menthol-derived chiral acyl chloride (derived from p-menthane-3-carboxylic acid) and 4-methoxyaniline (*p*-anisidine). Mechanistically, the primary amine of the electron-rich aniline acts as the nucleophile, attacking the highly electrophilic carbonyl carbon of the acyl chloride to displace the chloride leaving group. Sodium bicarbonate serves as an inorganic base in this Schotten–Baumann-type coupling to neutralize the hydrochloric acid byproduct, driving the reaction to completion. The reaction selectively forms the amide bond with absolute preservation of the three chiral centers on the cyclohexane ring, yielding acoltremon in an 82% chemical yield [293].

#### 2.5.13. Defactinib

**Figure 71:**
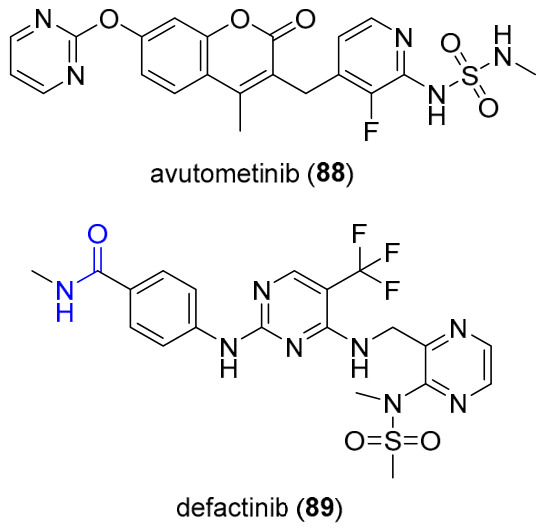
Chemical structure of avutometinib and defactinib.

Avutometinib (**88**) and defactinib (**89**) were approved together by the FDA as a combination therapy rather than as standalone drugs. On 8 May 2025, the agency granted accelerated approval to the co-packaged regimen marketed as Avmapki Fakzynja Co pack^®^, indicated for adult patients with KRAS-mutated recurrent low-grade serous ovarian cancer (LGSOC) who have previously received systemic therapy [294]. Only defactinib contains an amide group (Figure 71). The secondary amide building block was introduced intact rather than being generated de novo during the synthesis [253].

#### 2.5.14. Atrasentan

**Figure 72:**
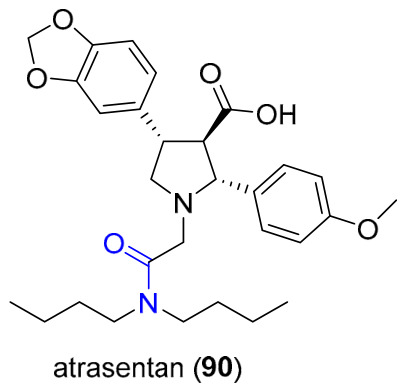
Chemical structure of atrasentan.

Atrasentan (**90**, Figure 72) received its first FDA approval on 2 April 2025, under the brand name Vanrafia^®^. The agency granted accelerated approval for its use in adult patients with primary immunoglobulin A nephropathy (IgAN) who are at risk of rapid disease progression, specifically to reduce proteinuria, a key surrogate marker of kidney damage [295]. Pharmacologically, atrasentan is a selective endothelin-A receptor antagonist, designed to block endothelin-mediated vasoconstriction and inflammatory signaling within the glomerulus, thereby reducing protein leakage into urine and potentially slowing structural kidney damage [296]. The secondary amide building block was introduced intact rather than being generated de novo during the synthesis [297].

#### 2.5.15. Gepotidacin

**Figure 73:**
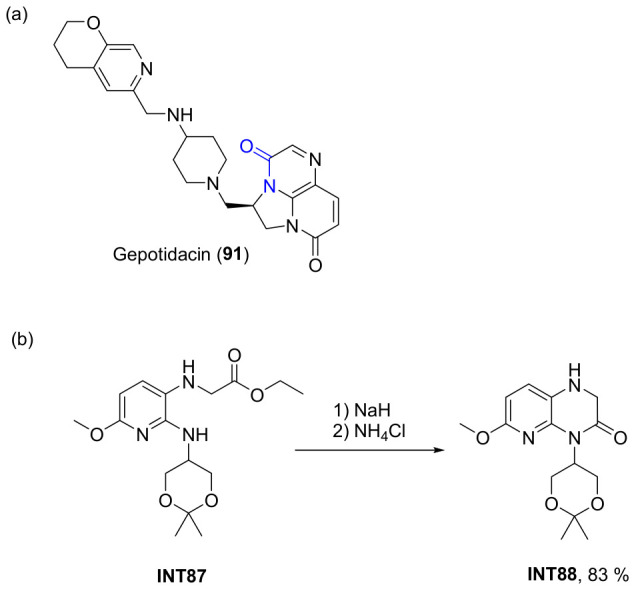
(**a**) Chemical structure of gepotidacin. (**b**) Preparation of **INT88**, used in the preparation of gepotidacin.

Gepotidacin (**91**, Figure 73a) was approved by the FDA on 25 March 2025, under the brand name Blujepa^®^. The approval covers the oral treatment of uncomplicated urinary tract infections (uUTIs) in female adults and pediatric patients aged 12 years and older who weigh at least 40 kg [298]. Gepotidacin is notable because it is a first-in-class triazaacenaphthylene antibiotic that inhibits bacterial DNA replication by targeting both DNA gyrase and topoisomerase IV at a site distinct from fluoroquinolones, helping retain activity against many drug-resistant pathogens [299]. The process functions as an intramolecular nucleophilic acyl substitution (lactamization). Sodium hydride acts as a strong base to deprotonate the more acidic secondary amine attached to the 1,3-dioxan-5-yl group. This generation of a highly nucleophilic amide anion triggers an intramolecular attack on the neighboring ethyl ester carbonyl carbon, displacing the ethoxide leaving group to close the new six-membered ring. Ammonium chloride is subsequently added as a mild acid quench to work up the reaction, successfully assembling the fused heterocyclic scaffold while leaving the acid-sensitive acetonide protective group intact [300].

#### 2.5.16. Vimseltinib

**Figure 74:**
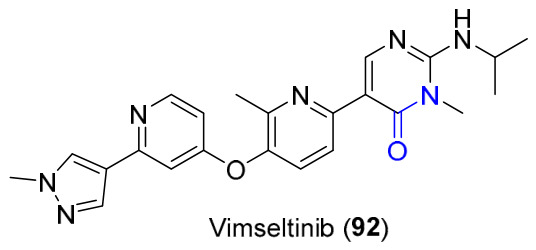
Chemical structure of vimseltinib.

Vimseltinib (**92**, Figure 74) received FDA approval on 14 February 2025, under the brand name Romvimza^®^, for the treatment of adult patients with symptomatic tenosynovial giant cell tumor (TGCT) in cases where surgical resection would likely cause severe morbidity or worsening functional limitation [301]. Vimseltinib is an oral colony-stimulating factor-1 receptor (CSF1R) switch-control kinase inhibitor that targets the signaling pathway responsible for recruiting inflammatory cells that drive TGCT growth, providing a targeted systemic therapy option for patients whose disease is not amenable to surgery [302].

#### 2.5.17. Suzetrigine

**Figure 75:**
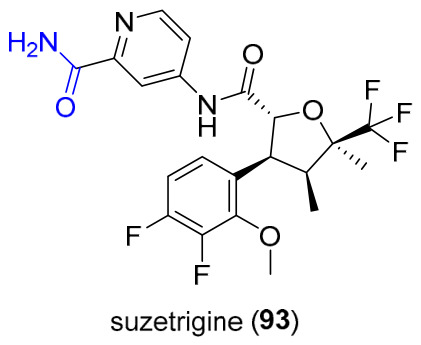
Chemical structure of suzetrigine.

On 30 January 2025, the FDA approved suzetrigine (**93**, brand name Journavx^®^, Figure 75) as the first non-opioid, selective NaV1.8 sodium channel inhibitor for treating moderate-to-severe acute pain in adults [303]. This oral medication targets peripheral pain signals without impacting central nervous system receptors, providing an alternative to traditional pain management. Suzetrigine’s approval emphasizes that Nav1.8 targeting may be a crucial tactic for managing moderate to severe acute pain [304]. At the moment, the number of Nav1.8 inhibitors in clinical trials is still low. With an IC_50_ of 0.7 nM and more than 31,000-fold selectivity against other NaV isoforms, VX-548 is a superbly selective inhibitor of NaV1.8. Important off-target effects on NaV1.4 (muscle contraction) and NaV1.5 (cardiac rhythm) are prevented by this selectivity [305]. Its structure and synthesis can be noted in [306,307].

#### 2.5.18. Elamipretide

**Figure 76:**
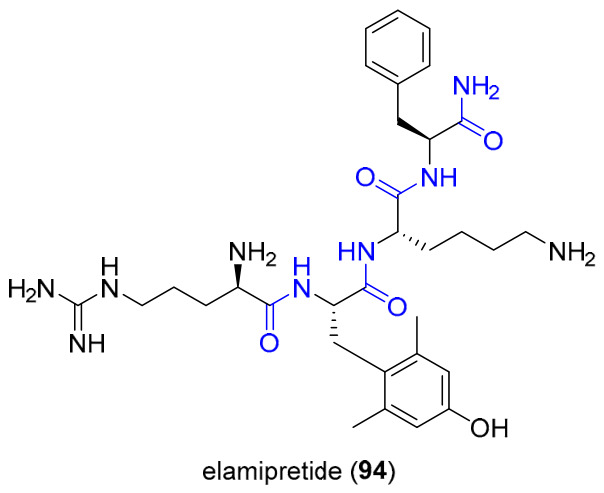
Chemical structure of elamipretide.

Approved on 19 September 2025, elamipretide (**94**, brand name Forzinity^®^, Figure 76) is a first-in-class, mitochondria-targeting tetrapeptide developed for treating diseases characterized by mitochondrial dysfunction [308]. In particular, it is the first approved treatment for Barth syndrome, a rare mitochondrial disease affecting primarily males [309]. Elamipretide has two positively charged amino acids (D-Arg and Lys), which allow it to preferentially target the inner mitochondrial membrane, which has a high negatively charged cardiolipin content [309].

## 3. Discussion

**Figure 77:**
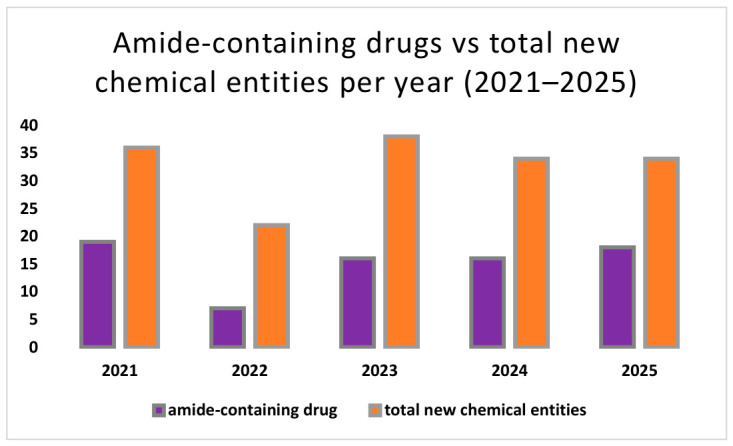
Bar chart showing the number of amide-containing drugs (MW < 1300 Da) vs. total new chemical entities per year (2021–2025).

The data shown in Figure 77 confirm that the amide bond remains a cornerstone of medicinal chemistry. In four out of the five years analyzed, nearly half (or more) of all New Chemical Entities (NCEs) contained at least one amide group. Only 2022 saw a significant drop in both total NCEs (22) and amide-containing entities (7). This general trend (for years 2021, 2023, 2024, and 2025) is likely due to the amide’s unique ability to provide structural rigidity and act as both a hydrogen bond donor and acceptor, which is critical for high-affinity binding to protein targets.

**Figure 78:**
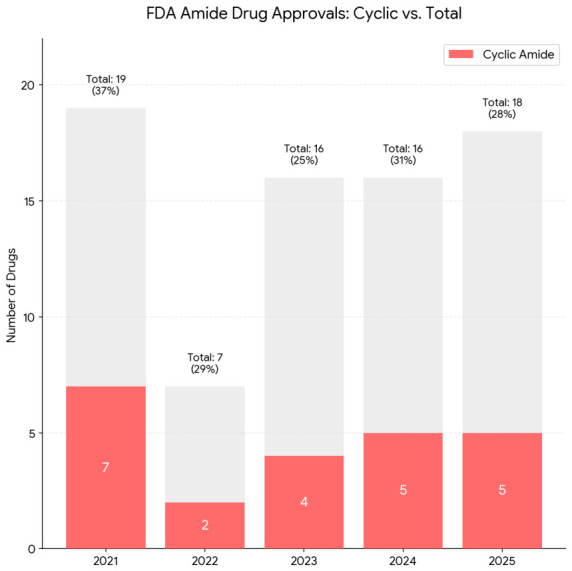
Graph showing FDA-approved drugs containing at least one cyclic amide (lactam) out of total amide-containing therapeutics.

In the evolving landscape of medicinal chemistry, the shift toward structural rigidity has become a hallmark of successful drug design. A longitudinal analysis of FDA approvals from 2021 to 2025 reveals that cyclic amides (lactams) consistently represent a significant portion of amide-containing therapeutics, ranging from 25% to 37% of annual approvals (Figure 78). This trend underscores a deliberate move away from flexible acyclic chains in favor of the enhanced metabolic stability and superior binding affinity offered by cyclic architectures. Seven out of a total (amide-containing) nineteen drugs in 2021 (approved by the FDA) contain at least one cyclic amide group. In 2022, two out of seven drugs contain at least one cyclic amide group. In 2023, 4 out of 16 drugs contain at least one cyclic amide group. In 2024, 5 out of 16 drugs contain at least one cyclic amide group. In 2025, 5 out of 18 drugs contain at least one cyclic amide group. The data show that cyclic amides (lactams) consistently represent a significant portion (roughly 25–30%) of amide-containing drugs approved by the FDA between 2021 and 2025. This trend can be rationalized through several key medicinal chemistry advantages that cyclic amides offer over their acyclic counterparts. The enhanced reactivity of β-lactams arises from the combined effects of significant ring strain and reduced amide resonance stabilization, as demonstrated in computational and spectroscopic studies [310]. Cyclic amide (pyridone/lactam) motifs represent privileged scaffolds in kinase inhibitor design, as their conformational rigidity and pre-organized hydrogen-bond donor–acceptor geometry enable consistent adenine-mimicking interactions with the hinge region—an effect exemplified by inhibitors such as Staurosporine, pyridone 6, and hydroxyfasudil, which all exploit cyclic amide groups to achieve high-affinity binding within the ATP pocket [311]. Conformational effects, such as distortion caused by ring strain or steric interactions, bridged amides, and specially designed cyclic systems that physically lock the amide into a non-planar geometry, are strategies used in organic chemistry to study and control amide reactivity by disrupting resonance [312].

**Figure 79:**
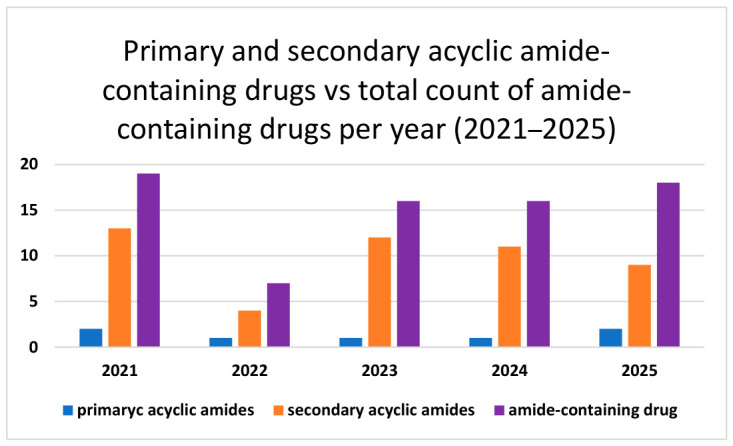
Number of drugs containing at least one amide functional group from 2021 to 2025, with separate counts for primary and secondary acyclic amides. Each compound was counted once per category if it contained at least one instance of the corresponding functional group.

The continued presence of cyclic amides is also driven by their dominance in specific therapeutic areas: Antibiotics; the lactam ring (a 4-membered cyclic amide) remains the most common structural motif in antibiotics like penicillins and cephalosporins [313]. In anticancer and rare diseases, there has been a growing trend in the approval of cyclic peptides and macrocycles (large rings containing multiple amides), which are used to target complex protein–protein interactions that smaller, linear molecules cannot [314]. The data shown in Figure 79 highlights that the number of amide-containing drugs remained relatively stable over the five-year period, fluctuating between 16 and 19 compounds per year (except for the year 2022). This suggests that amide functional groups continue to be consistently represented in approved or analyzed drug molecules. When distinguishing between amide subtypes, secondary acyclic amides were more prevalent than primary acyclic amides in every year examined. Secondary amides ranged from 4 to 13 occurrences, whereas primary acyclic amides appeared only 1–2 times per year, indicating that primary amides are comparatively rare in drug structures. It is important to note that each compound was counted once if it contained at least one instance of the functional group (e.g., at least one amide or at least one primary acyclic amide), rather than counting the total number of functional groups per molecule. Therefore, the values reflect compound prevalence, not functional group frequency. Primary acyclic amides were markedly underrepresented compared to secondary acyclic amides in all years analyzed. This trend is consistent with established medicinal chemistry principles: primary amides are more polar and often more susceptible to hydrolysis and enzymatic degradation. In contrast, secondary amides typically provide greater conformational rigidity, improved membrane permeability, and enhanced metabolic stability, which makes them more favorable in the design of orally bioavailable small-molecule drugs.

As can be seen from Table 1, the most represented therapeutic area is oncology, accounting for approximately 35–40% of all drugs, including subcategories such as targeted therapy, hematologic malignancies, CNS tumors, and diagnostics/radiopharmaceuticals. Other prominent areas include immunology/autoimmune (≈15%), infectious diseases (≈10%), and neurology/rare diseases (≈10%). Each year has a strong oncology presence, reflecting ongoing investment in cancer-targeted therapies and imaging agents. Subcategories like hematologic malignancies and solid tumor-targeted therapies are increasingly frequent from 2023 to 2025. There has been a rise in rare disease therapies: drugs for rare metabolic, mitochondrial, and neurodevelopmental disorders (e.g., omaveloxolone, trofinetide, elamipretide) appear predominantly in 2023–2025, highlighting a trend toward niche indications. Immunology has seen an expansion: autoimmune and allergy-targeted agents like rilzabrutinib, remibrutinib, and ritlecitinib show sustained growth, suggesting continued focus on immune-modulating therapies. Although fewer in number, key antiviral and antibacterial therapies (e.g., nirmatrelvir + ritonavir, rezafungin, and gepotidacin) highlight responsiveness to emerging infectious threats. Regarding diversification in therapeutic areas, other categories such as cardiology, women’s health/endocrine, ophthalmology, and diagnostic imaging reflect a growing diversity in the applications of amide-containing molecules beyond oncology and immunology.

**Figure 80:**
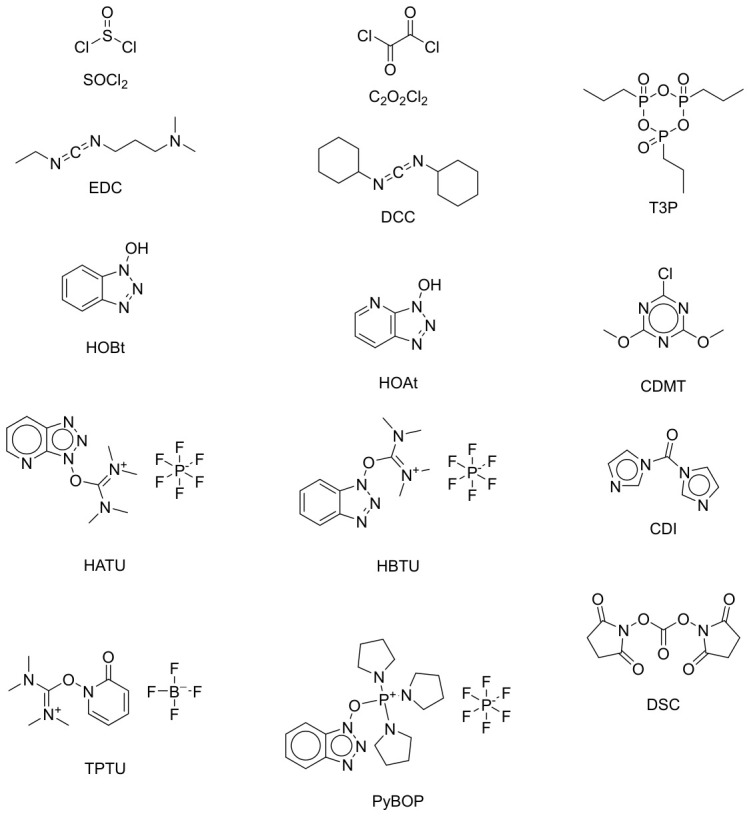
Main coupling reagents described in this article.

The ubiquity of the amide linkage in bioactive small molecules (seen in this article) has driven the optimization of diverse synthetic methodologies (Figure 80) within both discovery and process development phases. Classical approaches rely on the generation of highly electrophilic acyl halides via reagents such as thionyl chloride or oxalyl chloride. While highly efficient for sterically hindered systems, these conditions are frequently bypassed for complex scaffolds due to potential epimerization and functional group incompatibility. Conversely, the frequent deployment of acryloyl chloride highlights its indispensable specialized utility in appending acrylamide motifs acting as covalent warheads in targeted covalent inhibitors. To mitigate epimerization and establish milder reaction regimes, peptide coupling reagents are widely utilized. Carbodiimide architectures, notably EDC or DCC paired with suppressive nucleophilic additives like HOBt or HOAt, remain standard workhorses due to controllable kinetics and predictable profiles. For highly demanding or sterically constrained couplings, aminium/uronium and phosphonium systems such as HATU, HBTU, TPTU, and PyBOP are preferred. Specifically, HATU leverages a neighboring-group autocatalytic effect to deliver superior conversion rates in discovery chemistry. For scalable green chemistry applications and process development, alternatives like propylphosphonic anhydride (T3P) and triazine-based activators like 2-chloro-4,6-dimethoxy-1,3,5-triazine (CDMT) are increasingly favored, owing to their low epimerization risks, mild ambient conditions, and water-soluble byproducts that simplify downstream isolation. Furthermore, atom-economical reagents like carbonyldiimidazole (CDI) and *N*,*N*’-disuccinimidyl carbonate (DSC) provide clean pathways through transient imidazolide or active carbonate chemistry without requiring exogenous organic bases.

## 4. FDA-Approved Drugs Containing Amide in 2026

**Figure 81:**
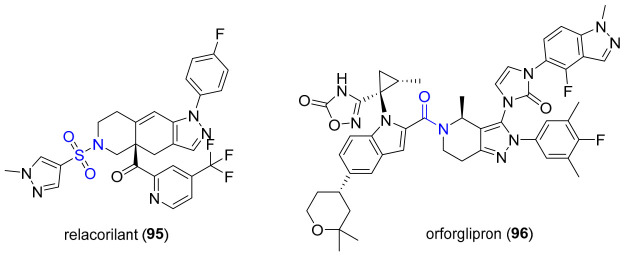
Chemical structure of relacorilant, a sulfonamide, and orforglipron (a tertiary amide).

The continuous wave of 2026 FDA approvals for small-molecule therapeutics (Figure 81) like relacorilant, a sulfonamide, and orforglipron (a tertiary amide) underscores the irreplaceable impact of the amide functional group in modern drug design, where its exceptional metabolic stability and precise geometric constraint act as the structural backbone needed to optimize binding affinity, enhance oral bioavailability, and successfully target complex disease pathways.

## 5. Concluding Remarks

Over the last five years (2021–2025), FDA-approved drugs containing amide functionalities have demonstrated a remarkable breadth of therapeutic applications, spanning oncology, immunology, neurology, infectious diseases, and rare metabolic disorders. Our analysis of 76 compounds highlights oncology as the dominant therapeutic area, reflecting sustained efforts in targeted cancer therapies, radiopharmaceutical diagnostics, and hematologic malignancy management. Concurrently, there is a notable increase in drugs addressing rare diseases and autoimmune conditions, signaling a strategic shift toward niche indications where unmet medical needs remain high. The emergence of antiviral and antibacterial amide-containing agents underscores the continued importance of amides in rapid response to infectious threats, exemplified by COVID-19 therapeutics and novel antibacterial compounds. The data also suggest an evolving landscape in pharmaceutical design, with amide moieties contributing to both bioavailability and solubility optimization, enabling effective drug delivery across diverse molecular classes. The consistent approval of amide-containing molecules across multiple therapeutic areas underscores their versatility and continued relevance in medicinal chemistry. Overall, these trends highlight the dual role of amide functionality in both pharmacological efficacy and favorable physicochemical properties, reinforcing its status as a core structural element in modern drug discovery. Ultimately, the evolution of amide bond synthesis, transitioning from classical, aggressive acyl halide reagents to highly refined, green, and atom-economical coupling frameworks, underscores the crucial bridge between innovative synthetic methodology and the efficient, sustainable delivery of modern therapeutic agents. Future research and development are likely to continue leveraging amide chemistry to address both common and rare diseases, while also optimizing solubility and pharmacokinetic profiles for enhanced clinical performance.

## Figures and Tables

**Table 1:** Amide-containing FDA-approved drugs (<1300 Da) grouped by therapeutic indication, primary indication and approval year.

Drug	Therapeutic Area	Primary Indication	Year
Pafolacianine	Oncology/Diagnostics	Intraoperative imaging of ovarian cancer	2021
Asciminib	Oncology	Chronic myeloid leukemia	2021
Mobocertinib	Oncology	EGFR-mutated non-small-cell lung cancer	2021
Sotorasib	Oncology	KRAS G12C-mutated non-small-cell lung cancer	2021
Piflufolastat F-18	Oncology/Diagnostics	PSMA PET imaging for prostate cancer	2021
Melphalan flufenamide	Oncology	Multiple myeloma	2021
Trilaciclib	Oncology/Supportive care	Chemotherapy-induced myeloprotection	2021
Tepotinib	Oncology	MET-altered non-small-cell lung cancer	2021
Avacopan	Immunology/Autoimmune	ANCA-associated vasculitis	2021
Belumosudil	Immunology	Chronic graft-versus-host disease	2021
Voclosporin	Immunology/Nephrology	Lupus nephritis	2021
Atogepant	Neurology	Migraine prevention	2021
Samidorphan	Psychiatry	Schizophrenia/bipolar disorder (combination therapy)	2021
Serdexmethylphenidate	Psychiatry/Neurology	ADHD	2021
Odevixibat	Hepatology/Rare Diseases	Progressive familial intrahepatic cholestasis	2021
Fosdenopterin	Metabolic/Rare Diseases	Molybdenum cofactor deficiency type A	2021
Difelikefalin	Nephrology	Pruritus in hemodialysis patients	2021
Finerenone	Cardio-renal/Metabolic	CKD in type 2 diabetes	2021
Cabotegravir	Infectious Diseases	HIV-1 treatment and prevention	2021
Futibatinib	Oncology	FGFR2-rearranged cholangiocarcinoma	2022
Olutasidenib	Oncology	IDH1-mutated acute myeloid leukemia	2022
Adagrasib	Oncology	KRAS G12C-mutated non-small-cell lung cancer	2022
Lutetium Lu 177 vipivotide tetraxetan	Oncology/Radiopharmaceutical	PSMA-positive metastatic prostate cancer	2022
Deucravacitinib	Immunology	Plaque psoriasis	2022
Mitapivat	Hematology/Metabolic	Pyruvate kinase deficiency	2022
Terlipressin	Gastroenterology/Critical Care	Hepatorenal syndrome	2022
Nirogacestat	Oncology	Desmoid tumors	2023
Capivasertib	Oncology	HR+/HER2- breast cancer	2023
Repotrectinib	Oncology	ROS1/TRK-positive NSCLC	2023
Fruquintinib	Oncology	Metastatic colorectal cancer	2023
Momelotinib	Oncology/Hematology	Myelofibrosis with anemia	2023
Lotilaner	Veterinary/Parasitology	Fleas and ticks in animals	2023
Ritlecitinib	Immunology/Autoimmune	Alopecia areata	2023
Nirmatrelvir + ritonavir	Infectious Diseases	COVID-19	2023
Fezolinetant	Women’s Health/Endocrine	Menopausal vasomotor symptoms	2023
Zavegepant	Neurology	Acute migraine	2023
Omaveloxolone	Neurology/Rare Diseases	Friedreich’s ataxia	2023
Daprodustat	Hematology/Nephrology	Anemia in chronic kidney disease	2023
Pirtobrutinib	Oncology/Hematologic malignancies	Mantle cell lymphoma	2023
Trofinetide	Neurology/Neurodevelopmental	Rett syndrome	2023
Rezafungin	Infectious Diseases	Invasive candidiasis	2023
Alyftrek	Respiratory/Rare Diseases	Cystic fibrosis	2024
Ensartinib	Oncology	ALK-positive non-small-cell lung cancer	2024
Iomeprol	Diagnostic Imaging	Iodinated contrast agent	2024
Revumenib	Oncology/Hematology	KMT2A-rearranged leukemia	2024
Sulopenem etzadroxil	Infectious Diseases	Complicated urinary tract infections	2024
Inavolisib	Oncology	PI3Kα-mutated breast cancer	2024
Levacetylleucine	Neurology/Rare Diseases	Cerebellar ataxia	2024
Lazertinib	Oncology	EGFR-mutated non-small-cell lung cancer	2024
Tovorafenib	Oncology	Pediatric low-grade glioma	2024
Ceftobiprole medocaril sodium	Infectious Diseases	Bacterial pneumonia and skin infections	2024
Danicopan	Hematology/Immunology	Paroxysmal nocturnal hemoglobinuria	2024
Vadadustat	Hematology/Nephrology	Anemia in chronic kidney disease	2024
Resmetirom	Hepatology/Metabolic	MASH	2024
Cefepime/enmetazobactam	Infectious Diseases	Complicated urinary tract infections	2024
Rilzabrutinib	Immunology/Autoimmune	Immune thrombocytopenia	2025
Remibrutinib	Immunology/Allergy	Chronic spontaneous urticaria	2025
Aficamten	Cardiology	Hypertrophic cardiomyopathy	2025
Sevabertinib	Oncology	HER2-mutant solid tumors	2025
Elinzanetant	Women’s Health/Endocrine	Menopausal vasomotor symptoms	2025
Brensocatib	Respiratory/Immunology	Bronchiectasis	2025
Zongertinib	Oncology	HER2-mutant cancers	2025
Dordaviprone	Oncology/CNS tumors	Diffuse midline glioma	2025
Delgocitinib	Dermatology/Immunology	Chronic hand eczema	2025
Sebetralstat	Immunology/Rare Diseases	Hereditary angioedema	2025
Sunvozertinib	Oncology	EGFR exon 20 insertion-positive NSCLC	2025
Acoltremon	Ophthalmology	Dry eye disease	2025
Defactinib	Oncology	Solid tumors	2025
Atrasentan	Nephrology	IgA nephropathy	2025
Gepotidacin	Infectious Diseases	UTIs/gonorrhea	2025
Vimseltinib	Oncology	Tenosynovial giant cell tumor	2025
Suzetrigine	Neurology/Pain	Acute pain	2025
Elamipretide	Metabolic/Rare Diseases	Primary mitochondrial myopathy	2025

## Data Availability

No new data were created or analyzed in this study. Data sharing is not applicable to this article.
